# The Role of Feed Restriction on DNA Methylation, Feed Efficiency, Metabolome, Biochemical Profile, and Progesterone Patterns in the Female Filial Generation (F1) Obtained From Early Feed Restricted Ewes (F0)

**DOI:** 10.3389/fphys.2021.779054

**Published:** 2021-12-27

**Authors:** Sonia Andrés, Ole Madsen, Olimpio Montero, Alba Martín, F. Javier Giráldez

**Affiliations:** ^1^Instituto de Ganadería de Montaña, CSIC-Universidad de León, León, Spain; ^2^Animal Breeding and Genomics, Wageningen University and Research, Wageningen, Netherlands; ^3^Instituto de Biología y Genética Molecular, CSIC, Valladolid, Spain

**Keywords:** feed efficiency, nutritional programming, feed restriction, progesterone, cholic acid, metabolomics, epigenetics

## Abstract

Deficient management of replacement animals in the farm during early developmental windows may promote adverse programming effects on reproductive traits and subsequent transmission to the next generation. In this sense, DNA methylation profiles allow researchers to decode epigenetic regulation mechanisms in mammals and identify novel candidate genes correlated with phenotype differences in both dams and offspring. Therefore, improving knowledge in the field of epigenetics and intergenerational effects caused by prenatal and postnatal early nutritional events (e.g., feed restriction) is crucial for refining strategies dedicated to animal breeding. In this study, we determined differences in the global blood methylation patterns, biochemical profile, and metabolome of ewe lambs (F1) born from either early feed restricted dams (F0-RES) or fed *ad libitum* (F0-ADL). Our data show that functional categories such as those related to cellular processes, phosphorylation, nervous system, immunity response, or reproductive function were enriched significantly in the F1-RES lambs due to differences in the methylation of genes in these categories. These F1-RES lambs did not show differences in feed efficiency during the replacement period but presented higher levels of insulin and triglycerides and reduced concentration of progesterone, whereas the metabolome profile demonstrated variations in the bile acid composition when compared with the F1-ADL lambs. Taken together, all these results suggest that intergenerational effects caused by early feed restriction of dams (F0) may persist in the F1 female lambs with negative consequences on genes involved in cellular processes and reproductive traits.

## Introduction

In most of the developed and developing countries, there is growing interest in improving the management of replacement and breeding sheep females to minimize the consequences of adverse environmental effects, thus enhancing reproductive performance (e.g., prolificacy and age at first lambing), productivity, and efficiency (Greenwood et al., 2017). In this sense, prenatal and early postnatal periods are critical developmental windows in which the phenotype of an organism is responsive to environmental factors (e.g., diet). For example, maternal and early postnatal feed restriction caused by different reasons (e.g., deficient management practices or insufficient milk produced under natural breeding conditions) may program offspring for restricted nutrient availability to enable a better adaptation to a poor nutritional environment. However, a mismatch between a low-nutrition environment to which the offspring is programmed, and later high availability of feed may lead to an increased predisposition to disease risk [e.g., metabolic syndrome; Brien et al., 2014]. This phenomenon has been referred to as the “developmental origins and health and disease” in humans (Barker and Osmond, 1986), or fetal and nutritional programming in many other different species such as rodents and lambs. Particularly in early feed restricted lambs, implications for important phenotypic traits such as marbling, fattening, feed efficiency (Santos et al., 2018b,c), or the onset of puberty (Santos et al., 2018a) have been demonstrated.

Some studies, mainly in humans, have demonstrated that these altered phenotypes can be transmitted across generations if the fetus and the germlines are affected (Schulz, 2010; Bhartiya et al., 2012). The field of epigenetics helps understand these changes that are inherited without altering DNA sequences or postnatal environment of the offspring and could be considered for (epi)genetic improvement of quantitative traits. Thus, DNA methylation at cytosine-guanine (CpG) nucleotides is an epigenetic mechanism that plays an important role in regulating gene transcription. A group of enzymes called DNA methyltransferases (DNMTs) catalyzes methylation of the cytosine residues in a CpG pair. Hypo-methylation or hyper-methylation of different CpG sites in the genome [e.g., gene promoters, intergenic sites, gene bodies, and CpG rich-regions (the so-called CpG islands)] relates to open or closed chromatin (euchromatin or heterochromatin), hereby increasing or reducing (even silencing) gene transcription, respectively (Chavatte-Palmer et al., 2018).

In sheep, it seems feasible to modify DNA methylation and gene expression by nutritional supplements (e.g., folate, methionine, and choline) administered to gestating (F0) dams (Anderson et al., 2012; Brien et al., 2014; Kitsiou-Tzeli and Tzetis, 2017; Chavatte-Palmer et al., 2018). Maternal under- and overnutrition of the gestating dam may also modify DNA methylation (Thompson et al., 2020), thus resulting in changes on F1 (fetus) and F2 (germ cells of the fetus that will promote F2 generation). However, it is not clear if nutritional restriction during the early life (suckling period) of ewe lambs kept for breeding purposes in the farm (F0) may promote epigenetic intergenerational transmission (e.g., endocrine function persistently altered) to the offspring (F1) through the germline (Bhartiya et al., 2012; Heard and Martienssen, 2014; Chavatte-Palmer et al., 2018). If this were the case, it would be crucial to identify those animals suffering from early feed restriction to avoid transmission of undesirable epigenetic marks to the progeny. Such an approach would definitely contribute to the development of breeding protocols providing higher productive, healthy, and efficient standards (Thompson et al., 2020). However, there is a scarcity of studies in livestock species due to the long generation intervals when compared to rodents (Greenwood et al., 2017) and totally lacking in sheep (Thompson et al., 2020).

Therefore, we hypothesized that postnatal programming events caused by early feed restriction (first month of life of F0) may affect postnatal oogenesis in ewe lambs, given the fact that *de novo* methylation of female germ cells only occurs after birth (Bhartiya et al., 2012; Chavatte-Palmer et al., 2018). We also hypothesized that these changes may be transmitted to the offspring (F1), modifying the physiological parameters of F1 over the lifespan. To test these hypotheses, this study was designed to describe the epigenetic marks, biochemical profiles, and the metabolome of the female offspring (F1) obtained from dairy ewes (F0) undergoing feed restriction during the suckling period. The epigenetic marks of the dams (F0) are also presented.

## Materials and Methods

### Care and Use of Animals

All handling practices involving animals followed the recommendations of the Directive 2010/63/EU of the European Parliament and of the Council on the protection of animals used for scientific purposes, and the experimental protocols were approved by the IGM-CSIC Animal Experimentation Committee (protocol number 2018-E04).

### Animals and Diets

All the details of F0 Assaf dams are fully described by Santos et al. (2018a, 2019). In brief, the replacement ewe lambs (F0) were divided into two groups, the first one being fed milk replacer (MR) *ad libitum* (F0-ADL) during the suckling period and the second one (F0-RES) being milk restricted (62.5% of *ad libitum* intake level) during the first 35 days of life. Thereafter, both groups were raised under similar post-weaning conditions (Santos et al., 2018a) and mated to obtain the female progeny used (F1-ADL and F1-RES).

The newborn Assaf female lambs (F1-ADL and F1-RES) were weighed (live body weight, LBW) and separated from the dams only after parturition and then bottle-fed colostrum for 2 days and commercial MR (Cordevit, Leches Maternizadas S.A., León, Spain) for 2 more days. Thereafter, all the animals were fed MR using an automatic feeder until they were 40 days old. All the female lambs were weighed twice a week throughout the suckling period. Ingredients and chemical composition of the MR are summarized in Supplementary Table 1.

All the lambs (F1) were weaned progressively during 7 days allowing free access to a starter feed for replacement lambs (Babymix, Inatega, Spain), barley straw and alfalfa hay; the accession to the automatic feeder of MR was allowed during only 1 h. Once the animals were weaned, they were moved to a single feedlot where they were fed *ad libitum* the same starter feed, barley straw and alfalfa hay supplied in separate feeders for 42 days. After this period, the ewe lambs were fed *ad libitum* a complete pelleted diet (CDP) formulated according to National Research Council (1985). Ingredients and chemical composition of the CPD administered are summarized in Supplementary Table 2.

### Animal Performance, Digestibility, Progesterone, and Biochemical Profile

When F1 lambs were 7 months old, all of them were individually penned for 8 weeks to measure CPD intake administered *ad libitum*; moreover, animals were weighed once a week to estimate feed efficiency. The protocols and conditions applied during this phase were identical to those previously published by Santos et al. (2018a). In brief, average daily weight gain (ADG, g/day) was estimated as the regression coefficient (slope) of LBW against time using the REG procedure of the SAS package (SAS Institute Inc., Cary, NC, USA). The feed conversion ratio (FCR) was obtained by dividing the feed intake per day by the ADG (g/day). Feed efficiency traits [e.g., residual feed intake (RFI) and residual intake and body weight gain (RIG)] were measured according to Giráldez et al. (2021).

When animals were 8.5 months old, feces were collected from each animal (rectal grab samples) for nine consecutive days at 08:00 a.m. before feed delivery in order to estimate the digestibility using acid insoluble ash (AIA) as an internal marker (van Keulen and Young, 1977).

Moreover, all the animals were blood sampled before weaning and then, weekly at 08:30 a.m. from the age of 5 months. Blood samples were collected by jugular venipuncture into tubes with no anticoagulant, which were allowed to clot in a water bath at 37°C for 30 min and then centrifuged at 3,520*g* for 16 min at 4°C. Serum samples were stored at −80°C until used for the analysis of progesterone concentration and the biochemical profile (albumin, aspartate aminotransferase (AST/GOT), gamma-glutamyl transpeptidase (GGT), beta-hydroxybutyrate (BHB), total bilirubin, Ca, Zn, Mg, ceruloplasmin, creatine kinase, creatinine, high-density lipoprotein (HDL), low-density lipoprotein (LDL), cholesterol, glucose, insulin, non-esterified fatty acids (NEFA), triglycerides, urea, protein, and globulin). Weekly progesterone measurements were performed on serum by sequential competitive immunoassay (Immulite^®^/Immulite^®^ 1000 Progesterone). The biochemical profile was measured at three time points (before weaning, 7 and 9 months old) using a clinical chemistry and turbidimetry analyzer Biosystems BA400 (Biosystems S.A., Barcelona, Spain).

### Reduced Representation Bisulfite Sequencing and Analyses

Blood samples were collected from five animals from each group (in total 20 samples; F1 lambs of 35 days old and F0 dams of 15 months old) by jugular venipuncture into tubes containing EDTA. DNA extraction was carried out using the QIAamp DNA Blood Mini Kit (Qiagen Cat# 51104). Samples were evaporated using a Concentrator Eppendorf plus system and then stored at −80°C. For one sample, the DNA extraction was of insufficient quality for reduced representation bisulfite sequencing (RRBS) library construction (F1-ADL group), so in this group, we had four samples. Data files can be accessed in the European Nucleotide Archive (ENA) using this link: http://www.ebi.ac.uk/ena/data/view/PRJEB47721.

Construction of RRBS libraries was carried out using the Ovation RRBS Methyl-Seq Kit from Tecan. In brief, 100 ng of high-molecular-weight DNA was digested with MspI, and size selected fragments of 20–500 bp were ligated with sequencing adaptors, treated with bisulfite, and amplified by PCR. The final libraries were quantitated with Qubit (ThermoFisher, MA, USA), and the average size was determined on a fragment analyzer (Agilent, CA, USA). The libraries were diluted to 10 nM and further quantitated by qPCR on a CFX Connect Real-Time qPCR System (Biorad, Hercules, CA, USA) for accurate pooling of barcoded libraries and maximization of the number of clusters in the flow cell.

The pooled barcoded shotgun libraries were loaded on an Illumina NovaSeq 6000 S1 lane for cluster formation and sequencing at the Roy J. Carver Biotechnology Center at the University of Illinois at Urbana-Champaign. They were sequenced for 100 nt from one side of the DNA fragments. The demultiplexed fastq data were generated using bcl2fastq version 2.20 Conversion Software (Illumina, San Diego, CA, USA), and adaptors were trimmed with Trim Galore using the default mode but without the RRBS setting as recommended by the NuGen protocol. Only reads longer than 20 bp were retained. The number of reads per sample varied between 36 million (M) reads to 51M with an average number of 45M reads [Supplementary Material 1 (Data and Methylation Stat)].

The RRBS data provided by the sequencing facility were trimmed with the custom Python script provided by NuGen (https://github.com/nugentechnologies/NuMetRRBS) to keep only the reads with the expected YGG bases at the beginning of the reads. Reads were aligned against the sheep reference genome (Oar_ramboulillet_v1.0) using BS-Seeker2 (version 2.1.8) (Guo et al., 2013). An RRBS genome was built from the reference genome with bs_seeker_build.py and the following parameters: –rrbs -l 10 -u 550 –aligner bowtie2. The trimmed reads were aligned against the RRBS genome with the bs_seeker2_align.py script using the following settings: -r -L 20 -U 550 -m 4 –aligner=bowtie2 –bt2-p 4 –bt2–local –bt2-N 1 –bt2-L 20. The CGmaptools (version 0.1.2) (Guo et al., 2018) convert bam2cgmap function was used to convert the BAM files into CpG methylation calls. CpG methylation calls were filtered to include only sites covered by 10 reads or more and subsequently converted into a Methylkit support format with a custom python script. The methylkit R package (version 1.8.1) (Akalin et al., 2012) was used for downstream analysis including differential methylation calls. Differential methylation calls were performed with the overdispersion function (overdispersion = MN) and a chi-square test for significant differences (test=Chisq) as suggested the best practices of bisulfite sequencing analysis (Wreczycka et al., 2017). Only position covered by 10 reads or more in all samples were included in the methylkit analysis, and the CpG in the 99.5th percentile of coverage were filtered out to remove potential biases caused by PCR duplicates. CpG calls on both strands were merged with the setting destrand=TRUE. A threshold of at least 25% differences in methylation and a false discovery rate of 0.05 (*q*-value < 0.05) were used to find significantly differential methylated sites between comparisons (F0-ADL vs. F0-RES and F1-ADL vs. F1-RES). The significantly differential methylation sites were functionally annotated by overlaying them with promoter regions (−2,000 to +200 around TSS—Ensembl annotation version 1.0.101) and gene body regions (+200 bp TSS to TTS) using bedtools (version 2.16.2). Differential methylated genes (DMGs) were defined as genes with significantly differential sites in the promoter and/or gene body. The Gene Ontology (GO) analyses were performed on the full dataset (significant hyper- and hypo-methylated sites) and on significant hypo- and hyper-methylated sites, respectively. GO biological processes and Kyoto Encyclopedia of Genes and Genomes (KEGG) pathway enrichment of the DMG comparisons were performed using the ShinyGO (version 0.61) webserver. The human GO and KEGG databases were used for these enrichment analyses applying an FDR < 0.05.

### Ultra-Performance Liquid Chromatography-Mass Spectrometry Analysis

When F1 lambs were 8 months old, all of them were blood sampled (before CPD distribution) by jugular venipuncture into tubes containing lithium-heparin, placed in iced water, and centrifuged at 3,520*g* for 16 min at 5°C. Then, plasma samples were stored at −80°C until metabolome analyses. For analyte extraction, 600 μl of cold acetonitrile (ACN) were added to 200 μl of plasma, the mixture was kept for 1 h at 4°C and later centrifuged, and 620 μl of the supernatant were withdrawn; this extract was further analyzed. An Acquity UPLC HSS T3 1.8 μm, 2.1 × 100 mm column with a pre-column (VanGuard 2.1 mm × 5 mm, 1.8 μm particle size) was used for the liquid chromatography (LC) analysis, which was performed in an acquity ultra-performance LC (UPLC^®^) from WATERS (Barcelona, Spain). Samples were analyzed with two different elution methods (7.5 μL of each sample were injected). In method 1, binary gradient elution was used where solvent A was methanol:water (2:8, v/v) + 0.1% formic acid and solvent B was 100% acetonitrile + 0.1% formic acid. The flow was 0.35 ml/min. The gradient elution of this method was initial, 99.9% A; 1 min, isocratic; 3.5 min, 20% A; 5.0 min, isocratic; 9.5 min, 0.1% A; 11.0 min, isocratic; and 14.0 min, 99.9% A (total run time was 15.0 min). In method 2, binary gradient elution was also used but where solvent A was methanol:water:formic acid (50:50:0.5, v/v/v) and solvent B was methanol:acetonitrile:formic acid (59:40:0.5, v/v/v). The flow was 0.30 ml/min. The gradient elution of this method was initial, 99.9% A; 1.0 min, isocratic; 2.5 min, 20% A; 4.0 min, isocratic; 5.5 min, 0.1% A; 8.0 min, isocratic; and 10.0 min, 99.9% A (total run time was 12.0 min). Samples were randomly distributed in the sample table to disperse error propagation. A sample pool was used as a quality control (QC) sample, which was injected at the onset, middle, and end of the sample set. Peak area values were later fitted according to QC values. Exogenous reserpine (3 μl of a 0.2 mg/ml reserpine solution) was also added to each sample as a reference compound, and chromatographic peak area was normalized to this of reserpine after integration. Analyte detection was conducted in a mass spectrometer SYNAPT HDMS G2 (WATERS, Barcelona, Spain) fitted with an electrospray ionization source (ESI, Z-spray^®^) and time of flight analyzer (ESI-QToF-MS). MS parameters were as follows: capillary 2.5 kV, source temperature 100°C, sampling cone 25, desolvation temperature 300°C, cone gas flow 35 L/h, desolvation gas flow 950 L/h, acquisition mass range 100–1,200 uma, and scan time 0.2 s. Leucine-enkephalin was used as a mass reference compound, and sodium formate was used for calibration according to the manufacturer indications. Samples were analyzed in positive and negative (only for method 1) ionization modes. The mass spectrometer was operated in an MS^E^ method, which includes a low energy function (full-scan equivalent) and a high energy function that continuously renders fragments of the base peak m/z.

MarkerLynx^®^ software (WATERS, Manchester, United Kingdom) was used to process the chromatographic and mass spectrometric (UPLC-MS) data. A three-dimensional data array comprising plasma sample (including blanks), retention time_*m*/*z* values (molecular features), and normalized signal intensity of the *m*/*z* value (scaled to Pareto variance) as variables was generated. Following, those *m*/*z* values present in the blank samples were manually excluded. The resulting data array was used later for principal component analysis (PCA) or partial least squares—discriminant analysis (PLS-DA). Tools of the SIMCA-P+ software package (Umetrics EZ info 2.0; Umea, Sweden) included in the Extended Statistics (XS) application of the MarkerLynx^®^ software were used for the multivariate statistical analysis (PCA and PLS-DA). The *m*/*z* values with a defined chromatographic peak were only accepted to potentially arise from any true compound. Differential metabolites were selected and tentatively identified by comparison of their *m*/*z* and elemental composition with freely available databases METLIN, HMDB, KEGG, ChEBI, and LipidMaps. The chromatographic peak area of the selected metabolites was later integrated using the QuantLynx^®^ application (Waters, Manchester, United Kingdom).

### Statistical Analysis

Data corresponding to animal performance of F1 lambs (ADG, RFI, RIG, and digestibility) were analyzed by one-way ANOVA using the GLM procedure of SAS (SAS Institute Inc., Cary, NC, USA) with the dietary treatment of F0 dams (*ad libitum* or restricted during early life) as the only source of variation. In all cases, the individual lamb was considered as the experimental unit. Significance was declared at *P* < 0.05.

Data corresponding to biochemical parameters measured at several growth stages (1, 7, and 9 months of life of F1 lambs) were analyzed as a repeated measures design using the MIXED procedure of SAS. Dietary treatment and growth stage were included in the model (the main effects of both factors and their interaction) as fixed effects. In all cases, the individual lamb was considered as the experimental unit and included in the model as a random effect. For each variable, the statistical model was fitted with different competing covariance structures, selected with the smallest value for Akaike and Bayesian criteria. The Tukey-Kramer multiple-comparison test was used to compare means when the interaction between dietary treatment and growth stage was significant.

## Results

### Animal Performance and Serum Progesterone Levels of F1 Lambs

Animal performance data can be found in Table 1. As can be observed, no differences in ADG during the suckling (212 vs. 206 g/day, *P* = 0.810) or replacement periods (111 vs. 126 g/day, *P* = 0.354) were observed between groups. Accordingly, no differences in the RFI, feed to gain ratio, digestibility parameters, or LBW (kg) were detected (*P* > 0.05). Moreover, considering that the first ovulation is reached when serum progesterone levels are above 0.4 ng/ml (Wright et al., 2002), no differences were observed in the age of puberty of F1 lambs (206 vs. 200 days, *P* = 0.518). However, the mean concentration of progesterone in the serum of F1-RES lambs was significantly lower when compared with the F1-ADL group (1.98 vs. 1.30 ng/ml, *P* = 0.018).

**Table 1:** Effect of early feed restriction of dams (F0) on the suckling (pre-weaning) and replacement performance of the female offspring (12 *ad libitum*, F1-ADL vs. 12 restricted, F1-RES).

	**F1-ADL**	**F1-RES**	**SED^a^**	***P*** **-value**
Birth LBW^b^, kg	4.34	4.81	0.807	0.159
Suckling period				
ADG suckling period, g/d	212	206	35.1	0.809
LBW at weaning, kg	14.48	13.99	1.302	0.597
Replacement period (RFI estimation)				
ADG, g/d	111	126	22.1	0.354
Initial LBW, kg	47.80	45.54	3.576	0.380
Final LBW, kg	55.10	53.89	3.128	0.590
DMI, g DM/animal day^−1^	1428	1463	97.4	0.614
RFI^c^, g DM animal^−1^ day^−1^	−12.0	11.0	47.70	0.500
Residual Weight Gain, g animal^−1^ day^−1^	0.37	−0.33	12.72	0.938
Residual intake and body weight gain	−0.16	0.15	1.099	0.696
Feed to gain ratio, g DMI/g ADG	13.65	13.214	2.450	0.804
DM digestibility, %	60.33	58.89	1.763	0.529
OM digestibility, %	62.52	61.12	1.768	0.545
Progesterone				
Days at first ovulation	206	200	9.445	0.518
Concentration of progesterone (ng/ml)	1.98	1.30	0.264	0.018

a *SE of the difference*.

b *LBW, live body weight*.

c *Residual feed intake (RFI) calculated using average daily weight gain (ADG) estimated by regression*.

### RRBS Methylation Analysis

The average level of methylation in the four groups varied between 53.4% and 53.7% [Supplementary Material 1 (Data and Methylation Stat)], and high methylation correlation was observed between all the samples (correlation 0.95–0.97, including F0 and F1; Supplementary Figure 1), so feed restriction did not cause detectable differences in global blood methylation patterns. This agrees with the lack of differences in feed efficiency (residual feed intake data) of both F0 and F1. Significantly methylated sites were assessed on 1.643.134 and 1.826.309 sites in F0 and F1, respectively. In total, 530 sites were found to be differentially methylated in F0 [Supplementary Material 2 (Significant Methylation Sites and Genes F0) and 1,588 sites in F1 [Supplementary Material 3 (Significant Methylation Sites and Genes F1)], respectively. Several promoters (TSS) and gene body (GB) sites showed significant differences in methylation when comparing both groups of lambs in both F0 (34 promoter sites in 25 genes and 142 gene body sites in 106 genes) and F1 (69 promoter sites in 61 genes and 429 gene body sites in 310 genes). All this information can be found in detail in Supplementary Material 2 (Significant Methylation Sites and Genes F0) and Supplementary Material 3 (Significant Methylation Sites and Genes F1)]. Two genes (*RIBC1* and *SUSD4*) in promoters and 13 genes (*AGBL1, ALDH1L2, CLEC16A, ERAL1, GNA12, HMCN2, RAPGEF1, RPTOR, SLC25A14, TENM1, TSC1, TSHR, ZNF407*) in the gene body matched both F0 and F1.

The functional gene enrichment analysis on all sites (TSS+GB) of F1 (offspring) showed differences in the methylation of genes for 238 GO biological processes (Supplementary Table 3), the 30 most significant terms having been compiled in Table 2. The main functional categories significantly affected in F1-RES lambs were related to cellular differentiation, morphogenesis, development, and proliferation, whereas many others were linked to neurons, axons, signaling, and communication processes. Other relevant enriched GO terms were cardiac muscle cell differentiation, cardiocyte differentiation, mating, response to estrogens, and immune system process (Supplementary Table 3). Separate analyses on significant hypo- and hyper-methylated sites in TSS and GB gave similar results but interestingly the hyper-methylated TSS (low methylation in F1-ADL group) resulted in enrichment in negative regulation of growth [Supplementary Material 4 (Gene List GO F1)].

**Table 2:** Thirty most significant GO enrichment terms from the list of differentially methylated genes in the female progeny (F1-RES) of early feed restricted ewes.

**Functional category**	**Enrichment FDR**	**^a^Genes in list**	**Total genes**
Movement of cell or subcellular component	0.0001116	52	2139
Cell projection organization	0.0001116	43	1589
Neuron differentiation	0.0001116	40	1412
Generation of neurons	0.0001505	42	1575
Plasma membrane bounded cell projection organization	0.0002188	41	1552
Cell differentiation	0.0002383	84	4459
Neuron projection development	0.0002383	31	1008
Cellular developmental process	0.0002383	87	4671
Neuron development	0.0003409	33	1154
Neurogenesis	0.0003441	42	1683
Regulation of signaling	0.0006905	75	3952
Regulation of cell communication	0.0007865	74	3903
Cell development	0.0011498	49	2230
Locomotion	0.0012228	44	1921
Cellular response to endogenous stimulus	0.0012228	36	1432
Regulation of neuron differentiation	0.0013293	22	665
Axon development	0.0013293	19	517
Positive regulation of metabolic process	0.0013589	71	3789
Response to endogenous stimulus	0.0014464	40	1704
Cellular component morphogenesis	0.0015798	31	1172
Cell morphogenesis	0.0016933	29	1067
Negative regulation of cell proliferation	0.0016933	23	737
Negative regulation of developmental process	0.0018194	28	1017
Cell-substrate adhesion	0.0018208	15	358
Regulation of response to stimulus	0.0018208	84	4820
Regulation of multicellular organismal development	0.0019242	46	2138
C-terminal protein amino acid modification	0.0019242	4	15
Nervous system development	0.0019361	51	2474
Response to growth factor	0.0027964	22	723
Regulation of cellular component biogenesis	0.0028863	27	1002

a *Number of genes differentially methylated in the functional category*.

For the dams undergoing feed restriction (F0-RES), the functional gene enrichment analyses did not provide any significant results when TSS and GB genes were combined but significant enrichment was achieved when TSS genes were used [Supplementary Material 5 (Gene List GO F0)]. In this latter, the functional categories found in F0 with differentially methylated genes were more involved with collagen biosynthesis and metabolic processes, together with other categories related to immune response (Supplementary Table 4).

### Metabolomic Profile

**Figure 1:**
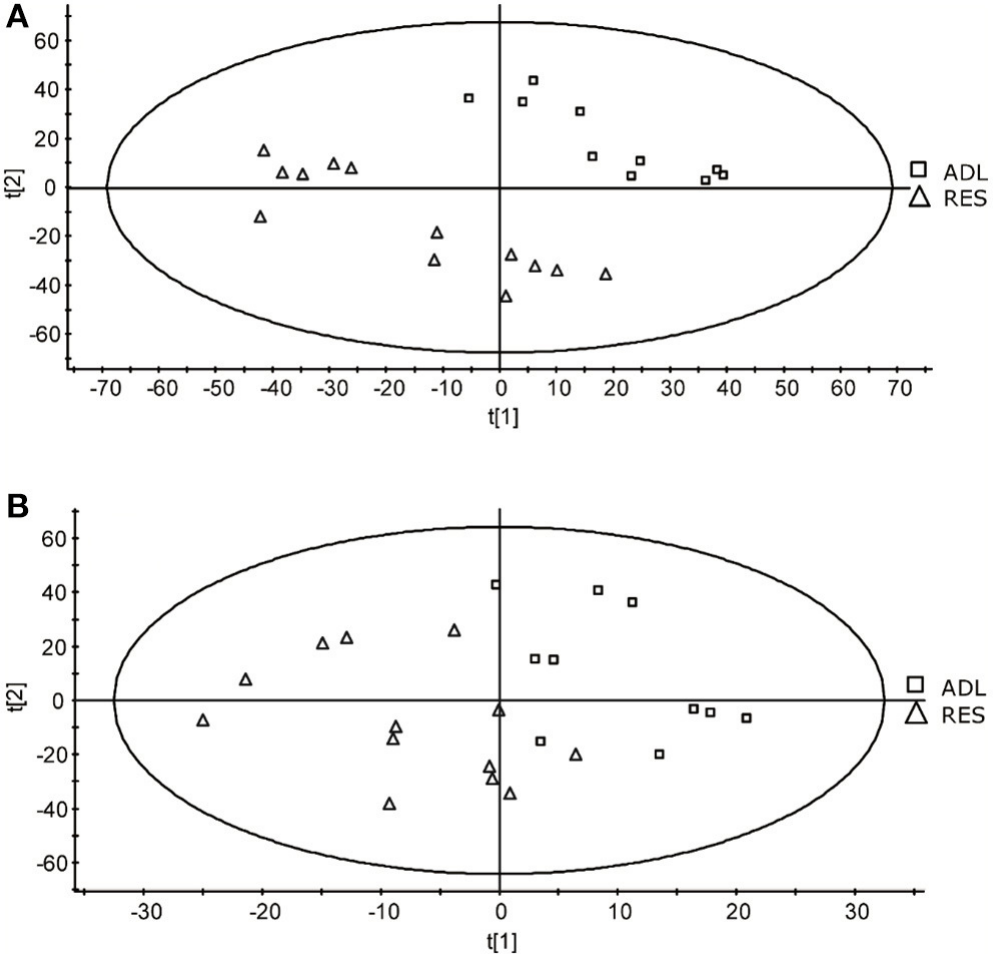
Score-plots obtained for the partial least squares - discriminant analysis (PLS-DA) of data from the methods 1 **(A)** and 2 **(B)** of the UPLC-MS analysis of plasma samples obtained from F1-ADL and F1-RES lambs (8-months old).

A comparison of typical chromatograms and averaged mass spectra obtained with the different methods is depicted in Supplementary Figure 2. The PLS-DA allowed to effectively separate the two treatment groups of 9-month F1 lambs (ADL and RES) according to mainly component 2 (Figure 1), resulting in a goodness-of-fit R2Y of 0.45 and a prediction ability Q2Y of 0.33 for component 1, and R2Y of 0.93 and a prediction ability Q2Y of 0.89 for component 2, when the data set of method 1 was used. Conversely, the PLS-DA treatment of the method 2 data set did not result in a so good separation as method 1 (Figure 1) but did give acceptable values of R2Y (0.54 and 0.68 for components 1 and 2, respectively) although very low-prediction ability (Q2Y = 0.1 and 0.02 for components 1 and 2, respectively).

Selected and tentatively identified metabolites are shown in Table 3, and their analytical data are shown in Supplementary Table 5. Some of them, mainly bile acid (BA)-related metabolites, were shown as potential biomarkers in both methods. Dihydroxy-cholan-24-oic acid and cholic acid (CA) were also pointed out as potential biomarkers in the PLS-DA of data from negative ionization (data not shown). However, a few compounds showed statistically significant differences (two-tailed *t*-test, *P* < 0.05) owing to the large dispersion of contents between samples. The contents of oxidized CA derivatives, glycocholic acid, and its reduced form glycodeoxycholate, along with taurocholic acid and taurodeoxycholic acid, were overaccumulated in the F1-RES group as compared with the F1-ADL group (Table 3). A number of lysophosphatidylcholines (LPCs) also showed higher contents in the RES group than in the ADL group, but only LPC(18:0) content was statistically significant (Table 3). An oxidized glycerolipid, namely, 1-hexadecanoyl-2-succinyl-sn-glycero-3-phosphate (PS-PA), which is described to be related to the immune system function (Hazen, 2008), was overaccumulated in F1-ADL lambs. Other metabolites with small molecular weights were also described as potential biomarkers contributing to explain the difference between groups, of which (homo)-2-citrate, trolamine, and indolelactic acid were overaccumulated in RES.

**Table 3:** List of identified metabolites (VIP score > 1.3) differentially accumulated in plasma samples (8 months old) from female lambs born from early feed restricted dams (F1-RES) when compared with those born from *ad libitum* dams (F1-ADL).

**Plasma metabolites**	**Method**	**VIP^a^ score**	**Log_**2**_FC^b^**	**Regulation^c^**
(Homo)2-citrate	1	5.28	0.23	Up*
Glycodeoxycolate	1	2.41	0.44	Up
Ketodeoxycholic acid	1/2	8.94/3.00	−0.19/−0.19	Down
Cholic acid	1/2	3.79/–	−0.17/0.15	Down/Up
Hydroxy-cholen-24-oic acid	1	4.02	−3.27	Down*
Dihydroxy-cholen-24-oic acid	1	2.43	−0.28	Down
Glycocholic acid	1/2	2.91/3.07	0.30/0.94	Up/Up
Triethanolamine (Trolamine)	1	3.94	0.17	Up
Indolelactic acid	1	4.87	0.09	Up
Tryptophan	1	2.76	−0.01	Down
C-10 Isoprenoid	2	1.71	−0.60	Down
Taurocholic acid 3-sulfate^d^	2	1.01	−0.65	Down*
Taurodeoxycholic acid	2	2.57	0.57	Up*
Taurocholic acid	2	2.49	0.22	Up*
LPC(17:1)^e^	2	2.66	0.06	Up
LPC(18:3)^f^	2	3.61	0.11	Up
LPC(18:3)^f^	2	1.52	−0.63	Down*
LPC(18:1)	2	5.29	−0.05	Down
LPC(22:5)	2	2.28	0.11	Up
LPC(22:4)	2	1.56	0.10	Up
LPC(18:0)	2	2.49	0.22	Up*
1-hexadecanoyl-2-succinyl-*sn*-glycero-3-phosphate (PS-PA)	2	1.32	−0.62	Down*

Method column refers to the ultra-performance liquid chromatography-mass spectrometry (UPLC-MS) and MarkerLynx methods used (for details see the “Materials and Methods” section). Significant differences (two-tailed t-test, P < 0.05) are indicated by *.

a *VIP, variable importance in projection*.

b *FC, fold change*.

c *Analyses were performed considering F1-RES vs. F1-ADL, therefore “up” means that the compound is over accumulated in F1-RES, as compared with the F1-ADL group*.

d *This compound was included in spite of having a VIP score lower than 1.3 since it showed statistically significant differences in the t-test. It should be acknowledged that the identification of this compound is questionable due to its elution time (Supplementary Table 5), but its m/z and elemental composition are pointed to in the databases METLIN and LipidMaps*.

e *LPC, lysophosphatidylcholine*.

f *They correspond possibly to two stereoisomers as the elution time was different enough (3.57 and 4.23 min) to be an analytical artifact*.

### Biochemical Profile

The effects of maternal (F0) early feed restriction on the biochemical profile of F1 female offspring during the artificial rearing (before weaning) and replacement phase (7 and 9 months old) are presented in Table 4. Insulin (9.06 vs. 13.36 μUI/ml, *P* = 0.033) was increased in F1-RES lambs, and triglycerides showed a trend toward increased values in F1-RES (23.63 vs. 29.66 mg/dL, *P* = 0.065). On the contrary, albumin (41.34 vs. 39.74 g/L, *P* = 0.048) and creatinine (0.98 vs. 0.94 mg/dL, *P* = 0.046) were decreased in F1-RES lambs. No changes *(P* > 0.05) were observed for the rest of the biochemical parameters (e.g., AST/GOT, GGT, BHB, total bilirubin, Ca, Zn, Mg, ceruloplasmin, creatine kinase, HDL, LDL, total cholesterol, glucose, NEFA, urea, protein, and globulin).

**Table 4:** Biochemical parameters of the female progeny obtained from early feed restricted ewes (F1-RES) and ewes fed *ad libitum* (F1-ADL).

			**Growth stage**					***P*** **-values**		
	**F1-ADL**	**F1-RES**	**1st month**	**7th month**	**9th month**	**SED^1^**	**SED^2^**	**Group**	**Day**	G*Day
Albumin (g/L)	41.34	39.74	34.07^a^	43.41^b^	44.14^b^	0.760	0.579	0.048	<0.001	0.014
AST/GOT^3^ (U/L)	127.6	129.5	87.06^a^	164.7^b^	133.9^b^	15.66	15.15	0.903	<0.001	0.969
GGT^4^ (U/L)	82.02	77.97	102.4^b^	67.10^a^	55.50^a^	8.354	6.860	0.633	<0.001	0.919
BHB^5^ (mmol/L)	2.52	3.07	1.40^a^	3.23^b^	3.76^b^	0.439	0.331	0.216	<0.001	0.327
Total bilirubin (mg/dL)	0.48	0.55	0.78^c^	0.44^b^	0.33^a^	0.045	0.053	0.110	<0.001	0.266
Ca (mg/dL)	10.55	10.54	10.69^a^	10.58^ab^	10.37^b^	0.138	0.210	0.935	0.030	0.445
Zn (ug/dL)	93.42	90.88	98.96	90.03	87.84	4.429	5.425	0.572	0.094	0.623
Mg (mg/dL)	2.33	2.37	2.29^a^	2.36^ab^	2.40^b^	0.058	0.041	0.441	0.028	0.463
Ceruloplasmin (mg/dL)	2.45	2.78	2.24^a^	2.17^a^	3.45^b^	0.224	0.273	0.146	<0.001	0.884
Creatine kinase (U/L)	274.0	302.8	259.0	354.3	251.9	81.48	99.79	0.727	0.523	0.487
Creatinine (mg/dL)	0.98	0.94	0.85^a^	1.02^b^	1.01^b^	0.020	0.014	0.046	<0.001	0.076
HDL^6^ (mg/dL)	62.89	59.94	70.76^c^	52.48^a^	61.00^b^	3.911	2.531	0.459	<0.001	0.045
LDL^7^ (mg/dL)	22.90	21.01	23.48	21.26	21.14	1.957	1.329	0.344	0.106	0.075
Cholesterol (mg/dL)	93.78	87.58	106.8^b^	81.03^a^	84.13^a^	6.086	3.216	0.320	<0.001	0.005
Glucose (mg/dL)	77.51	75.77	90.89^b^	69.22^a^	69.81^a^	3.321	3.021	0.606	<0.001	0.089
Insulin (uUI/mL)	9.06	13.36	7.92^a^	12.31^b^	13.40^b^	1.887	1.657	0.033	0.007	0.651
NEFA^8^ (mmol/L)	0.16	0.21	0.29^b^	0.12^a^	0.14^a^	0.033	0.041	0.205	<0.001	0.460
Triglycerides (mg/dL)	23.63	29.66	36.20^b^	22.85^a^	20.89ª	3.116	3.673	0.065	<0.001	0.484
Urea (mg/dL)	31.95	31.23	23.61^a^	34.61^b^	36.56^b^	1.494	1.565	0.634	<0.001	0.449
Protein (g/L)	62.66	62.13	54.08^a^	66.82^b^	66.28^b^	0.778	0.824	0.498	<0.001	0.913
Globulin (g/L)	21.32	22.38	20.01^a^	23.41^b^	22.14^b^	0.964	0.753	0.281	<0.001	0.090

1 *SED, SE of the difference to compare experimental groups*.

2 *SED, SE of the difference to compare days*.

3 *AST/GOT, aspartate aminotransferase*.

4 *GGT, gamma-glutamyl transpeptidase*.

5 *BHB, beta-hydroxybutyrate*.

6 *HDL, high-density lipoprotein*.

7 *LDL, low-density lipoprotein*.

8 *NEFA, nonesterified fatty acid*.

a−d *Different superscripts in the same line indicate statistical differences (P < 0.05) between groups (F1-RES vs. F1-ADL)*.

## Discussion

Epigenetic marks in the germline of early feed restricted lambs (F0) may be responsible for the intergenerational inheritance of environmentally induced altered phenotypes to the offspring (F1). In this study, more differential DNA methylated sites in both gene promoters (TSS) and gene bodies (GB) were detected in pairwise comparison of the F1 generation compared with F0. This seems to indicate that feed restriction during the suckling phase of ewe lambs (F0) might have influenced greatly the next generation (F1) due to the effects on the germline during the postnatal oogenesis of F0 (Bhartiya et al., 2012; Virant-Klun, 2015). In any case, our results in F1 agree with Sandovici et al. (2011), who observed a decreased expression of *HNF4A* (a gene encoding the hepatocyte nuclear factor 4 alpha that was hyper-methylated in F1-RES) in the offspring of malnourished mothers and an increased risk of type 2 diabetes. However, we could not observe differences in the methylation of *HNF4A* in F0 dams; nevertheless, epigenetic marks in F1 are not fully comparable to those in F0 due to the different ages of the animals sampled for RRBS analyses (15 months old for F0 and 35 days old for F1). This circumstance limits the direct comparison between both generations due to dynamic DNA methylation with aging.

However, the hypothesis that postnatal early feed restriction of ewe lambs (F0) can promote intergenerational transmission of epigenetic marks to the offspring (F1) is also supported by the GO enrichment analysis, where GO terms, e.g., mating, response to estrogens, and immune system process (Supplementary Table 3) were observed in F1-RES lambs. At this point, it is pertinent to remember that F0 dams presented upregulation of many genes involved in metabolic pathways (e.g., steroid and steroid hormone biosynthesis, primary BA biosynthesis, and bile secretion) just after the feed restriction period (Santos et al., 2019). Later on, during the replacement phase, animals showed long-term effects caused by early feed restriction such as altered steroid hormone biosynthesis, modified vitamin D metabolites, retarded puberty, pro-inflammatory conditions, and impaired liver function (Frutos et al., 2018a; Santos et al., 2018a). According to the transcriptome profile observed for these lambs (Santos et al., 2019), most of these effects might be explained, at least partially, by alterations in cholesterol metabolic pathways, given the fact that cholesterol is the precursor of steroid hormones (e.g., progestagens, glucocorticoids, mineralocorticoids, androgens, and estrogens) and BAs (Watanabe et al., 2004). Therefore, considering the results observed in this study, some of these features in F0 dams might have been transferred to the progeny (F1) by epigenetic marks, with consequences at different functions, as explained below.

### Energy and Protein Metabolism

Primary BAs (e.g., CA and chenodeoxycholic acid) are synthesized in the liver cells when cholesterol is oxidized *via* cytochrome P-450 and transformed in the intestine by bacteria into secondary BAs (e.g., glycocholic acid). Therefore, the increments of glycocholic acid and glycodeoxycholate, together with the increased amounts of indolelactic acid in the metabolic profile of F1-RES might also be related to differences in the gut microbiome, which at the same time may be affected by host genetics suggesting some degree of heritability of the gut microbiota (Li et al., 2019).

In any case, BAs act as important signaling molecules that regulate lipid, glucose, and energy metabolism/expenditure. For example, CA prevents elevated serum triglycerides in mouse models of hypertriglyceridemia (Watanabe et al., 2004; Ippagunta et al., 2018). Accordingly, the reduced levels of CA (and its derivatives) detected in the plasma of the F1-RES group might be responsible for the higher levels of triglycerides observed in this group of lambs. Interestingly, CA increases energy expenditure and insulin sensitivity in several types of cells (Sonne et al., 2014), so the high levels of insulin observed in F1-RES lambs are aligned with the low concentration of CA. In other words, low levels of CA, which were probably caused by transmission from F0, might be related to metabolic syndrome features in F1-RES lambs (Frutos et al., 2018b; Santos et al., 2018b).

Moreover, mitogen-activated protein (MAP) kinases participate in signal transduction pathways mediating in a wide variety of cellular processes, such as proliferation, differentiation, transcription regulation, and development. Therefore, the hyper-methylation in F1-RES of the *MAP2K3* and *MAPK10* genes caused the significant enrichment of many functional categories of genes (e.g., protein phosphorylation, regulation of protein metabolic process, …) which may play critical roles in the regulation of these cellular processes. Low levels of albumin and creatinine in F1-RES lambs, which are indicators of impaired liver function, might be related to these marks.

### Cholesterol Metabolism and Inflammation

Cholic acid also mediates a negative feedback regulation of BA synthesis, so it is plausible that the lower CA levels are related to increased amounts of cholesterol diverted toward BA synthesis (and probably BA secretion) in F1-RES (Li-Hawkins et al., 2002). In fact, even though the levels of cholesterol (total HDL and LDL) were not altered, total bilirubin (secreted in the bile together with BA) showed higher numerical values in F1-RES lambs (*P* = 0.110). As far as epigenetic marks are concerned, the gene encoding apolipoprotein A1 (*APOA1*), which is the major protein component of HDL particles in plasma and is often used as a biomarker for the prediction of cardiovascular diseases (typically linked to metabolic syndrome), was hyper-methylated in F1-RES. Another gene differentially methylated in F1-RES lambs that might promote differences in cholesterol metabolism and inflammatory profile of the offspring is *IL17REL* (with both hyper- and hypo-methylated sites in F1-RES). This gene encodes the receptor for IL17, a pro-inflammatory cytokine. However, given the lack of functional categories significantly enriched and specifically related to inflammation, together with the rich-forage diet (not acidogenic) administered to F1 during the replacement phase, we have no evidence to suggest an increased pro-inflammatory profile in the F1-RES lambs.

### Fertility and Progesterone Concentration

The main function of the corpus luteum is to synthesize and secrete progesterone, which regulates the duration of the estrous cycle and maintains pregnancy (when produced by placenta) in many species, including sheep. However, this study highlights lower progesterone concentrations together with increased levels of insulin and triglycerides in F1-RES lambs compared with the F1-ADL. This is similar to what happens in women suffering the polycystic ovary syndrome (PCOS), a complex multigenic disorder with strong epigenetic and environmental influences (including diet; Azziz, 2018; Escobar-Morreale, 2018) and characterized by a low-progesterone concentration during the luteal phase that may be responsible for infertility and early pregnancy losses (Meenakumari et al., 2004). The reasons for low progesterone synthesis during the luteal phase in PCOS women are not totally understood, but many patients with PCOS have features of metabolic syndrome (similarly to our F1-RES lambs) such as visceral obesity, dyslipidemia, and insulin resistance with compensatory hyperinsulinemia, which may adversely affect follicular development and ovulation by increasing intraovarian androgen production (Meenakumari et al., 2004).

Moreover, DNA methylation mediates cell responses to physiological endocrine signals, so epigenetic changes produced during development might affect persistently endocrine function (García-Carpizo et al., 2011). In addition, BAs also have hormonal actions throughout the body, particularly through the farnesoid X receptor and GPBAR1 (also known as TGR5), playing an important role in the regulation of intrafollicular steroid production (Nagy et al., 2019), so the different composition of BAs observed in F1-RES might be partially responsible for the low-progesterone concentration. In any case, two functional categories of genes related to reproduction (e.g., mating and response to estrogens) were significantly enriched in F1-RES lambs.

### Neurogenesis, Animal Behavior, and Response to Stimuli

Previous studies have shown that dysregulation of thyroid hormones caused by the maternal restriction of the diet may negatively affect the welfare of newborn animals and cause thyroid-mediated alterations of brain development in later life (Jenkinson et al., 2012; Viguié et al., 2020). Similar effects have been observed with the release of glucocorticoids (e.g., cortisol) by stressed mothers, which can cross the placenta to affect the developing offspring and promote epigenetic alterations in the brain and changes in the development of neurogenesis (Bertram et al., 2008; Sinclair et al., 2016). These epigenetic effects promoted by the early environment (e.g., prenatal stress or feed restriction) appear to be universal in mammals and may cause modified responsiveness to different stimuli and altered behavior changes (Brien et al., 2014). Thus, in this study, the differential methylation of genes in pathways related to neurogenesis/synapsis observed in F1-RES corroborates that epigenetic marks transferred to the offspring may be affecting the neurological processes of the offspring. In agreement with this statement, the metabolomic profile of F1-RES lambs points toward modified LPC levels (most of them overaccumulated in F1-RES, Table 4) as one of the underlying mechanisms causing neurogenesis alterations (Law et al., 2019).

### Ion Transport

Other metabolic pathways detected by the enrichment analysis in F1-RES lambs were those related to ion transport. Mineralocorticoids (mainly aldosterone, which is produced in the adrenal cortex using cholesterol as a precursor) act on the distal tubules of the kidney to increase the reabsorption of Na^+^ and the excretion of K^+^ and H^+^, thus increasing blood volume and pressure. In this regard, Zhang et al. (2011) demonstrated that maternal undernutrition only slightly prior to conception provoked both, increments of the adrenal gland weight in the F1 generation and differences in DNA methylation at the IGF2/H19 imprinted genes.

## Conclusion

In summary, our study shows that MR restriction of dams (F0) at 62.5% of *ad libitum* intake did not promote differences in feed efficiency traits of the offspring (F1) during the replacement phase. However, these animals presented features related to the metabolic syndrome, together with reduced mean concentrations of progesterone and a marked different profile of BAs. Therefore, our data support the hypothesis that changes caused by early developmental conditions of the dams can be transmitted to the female offspring (F1) through epigenetic marks. Understanding how early disturbances in ewe lambs are linked to later-life disease/transmission to the offspring/reproductive traits may suggest new ways to increase the profitability of the farms.

## Data Availability Statement

The RRBS data used in this study is available by the following ENA accession number PRJEB47721 (http://www.ebi.ac.uk/ena/data/view/PRJEB47721).

## Ethics Statement

The animal study was reviewed and approved by IGM-CSIC Animal Experimentation Committee (protocol number 2018-E04).

## Author Contributions

SA and FG contributed to conception and design of the study. OMad listed the differentially methylated genes. OMon carried out the metabolic assay. FG performed the statistical analysis. SA wrote the first draft of the manuscript. AM revised the previous literature and wrote sections of the manuscript. All authors contributed to manuscript revision, read, and approved the submitted version.

## Funding

This study was funded by Ministerio de Ciencia e Innovación (RTI2018-099329-B-I00, MCIN/AEI/10.13039/501100011033, FEDER, Una manera de hacer Europa). AM gratefully acknowledges the receipt of a predoctoral grant (PRE2019-089288) from Ministerio de Ciencia e Innovación (MCIN/AEI/10.13039/501100011033, El FSE invierte en tu futuro).

## Conflict of Interest

The authors declare that the research was conducted in the absence of any commercial or financial relationships that could be construed as a potential conflict of interest.

## Publisher's Note

All claims expressed in this article are solely those of the authors and do not necessarily represent those of their affiliated organizations, or those of the publisher, the editors and the reviewers. Any product that may be evaluated in this article, or claim that may be made by its manufacturer, is not guaranteed or endorsed by the publisher.

## References

[B1] Akalin A. Kormaksson M. Li S. Garrett-Bakelman F. E. Figueroa M. Melnick A.. (2012). MethylKit: a comprehensive R package for the analysis of genome-wide DNA methylation profilese. Genome Biol. 13:R87. 10.1186/gb-2012-13-10-r87 23034086 PMC3491415

[B2] Anderson O. S. Sant K. E. Dolinoy D. C. (2012). Nutrition and epigenetics: an interplay of dietary methyl donors, one-carbon metabolism and DNA methylation. J. Nutr. Biochem. 23, 853–859. 10.1016/j.jnutbio.2012.03.003 22749138 PMC3405985

[B3] Azziz R. (2018). Polycystic ovary syndrome. Obstet. Gynecol. 132, 321–336. 10.1097/AOG.0000000000002698 29995717

[B4] Barker D. J Osmond C. (1986). Infant mortality, childhood nutrition, and ischaemic heart disease in England and Wales. Lancet 1, 1077–1081. 10.1016/S0140-6736(86)91340-1 2871345

[B5] Bertram C. Khan O. Ohri S. Phillips D. I. Matthews S. G. Hanson M. A. (2008). Transgenerational effects of prenatal nutrient restriction on cardiovascular and hypothalamic-pituitary-adrenal function. J. Physiol. 586, 2217–2229. 10.1113/jphysiol.2007.147967 18292131 PMC2465204

[B6] Bhartiya D. Sriraman K. Gunjal P. Modak H. (2012). Gonadotropin treatment augments postnatal oogenesis and primordial follicle assembly in adult mouse ovaries? J. Ovarian Res. 5:32. 10.1186/1757-2215-5-32 23134576 PMC3616927

[B7] Brien F. D. Cloete S. W. P. Fogarty N. M. Greeff J. C. Hebart M. L. Hiendleder S.. (2014). A review of the genetic and epigenetic factors affecting lamb survival. Anim. Prod. Sci. 54, 667–693. 10.1071/AN1314028948418

[B8] Chavatte-Palmer P. Velazquez M. A. Jammes H. Duranthon V. (2018). Review: epigenetics, developmental programming and nutrition in herbivores. Animal 12, S363–S371. 10.1017/S1751731118001337 30139395

[B9] Escobar-Morreale H. F. (2018). Polycystic ovary syndrome: definition, aetiology, diagnosis and treatment. Nat. Rev. Endocrinol. 14, 270–284. 10.1038/nrendo.2018.24 29569621

[B10] Frutos J. Andrés S. Trevisi E. Benavides J. Santos N. Santos A.. (2018a). Moderated milk replacer restriction of ewe lambs alters gut immunity parameters during the pre-weaning period and impairs liver function and animal performance during the replacement phase. Anim. Feed Sci. Technol. 243, 80–89. 10.1016/j.anifeedsci.2018.07.009

[B11] Frutos J. Andrés S. Trevisi E. Yáñez-Ruiz D. R. López S. Santos A.. (2018b). Early feed restriction programs metabolic disorders in fattening Merino lambs. Animals 8:83. 10.3390/ani8060083 29857527 PMC6025537

[B12] García-Carpizo V. Ruiz-Llorente L. Fraga M. Aranda A. (2011). The growing role of gene methylation on endocrine function. J. Mol. Endocrinol. 47, R75–89. 10.1530/JME-11-0059 21803863

[B13] Giráldez F. J. Santos N. Santos A. Valdés C. López S. Andrés S. (2021). Fattening lambs with divergent residual feed intakes and weight gains: unravelling mechanisms driving feed efficiency. Anim. Feed Sci. Technol. 273:114821. 10.1016/j.anifeedsci.2021.114821

[B14] Greenwood P. Clayton E. Bell A. (2017). Developmental programming and beef production. Anim. Front. 7, 38–47. 10.2527/af.2017-0127

[B15] Guo W. Fiziev P. Yan W. Cokus S. Sun X. Zhang M. Q.. (2013). BS-Seeker2: a versatile aligning pipeline for bisulfite sequencing data. BMC Genomics 14:774. 10.1186/1471-2164-14-774 24206606 PMC3840619

[B16] Guo W. Zhu P. Pellegrini M. Zhang M. Q. Wang X. Ni Z. (2018). CGmapTools improves the precision of heterozygous SNV calls and supports allele-specific methylation detection and visualization in bisulfite-sequencing data. Bioinformatics 34, 381–387. 10.1093/bioinformatics/btx595 28968643 PMC6454434

[B17] Hazen S. L. (2008). Oxidized phospholipids as endogenous pattern recognition ligands in innate immunity. J. Biol. Chem. 283, 15527–15531. 10.1074/jbc.R700054200 18285328 PMC2414290

[B18] Heard E. Martienssen R. A. (2014). Transgenerational epigenetic inheritance: myths and mechanisms. Cell 157, 95–109. 10.1016/j.cell.2014.02.045 24679529 PMC4020004

[B19] Ippagunta S. M. Kharitonenkov A. Adams A. C. Hillgartner F. B. (2018). Cholic acid supplementation of a high-fat obesogenic diet suppresses hepatic triacylglycerol accumulation in mice via a fibroblast growth factor 21-dependent mechanism. J. Nutr. 148, 510–517. 10.1093/jn/nxy022 29659970

[B20] Jenkinson C. M. C. Earl A. K. Kenyon P. R. Blair H. T. (2012). Effects of maternal nutrition during pregnancy on fetal growth and maternal constraint in sheep. Anim. Prod. Sci. 52, 524–532. 10.1071/AN1124517667859

[B21] Kitsiou-Tzeli S. Tzetis M. (2017). Maternal epigenetics and fetal and neonatal growth. Curr. Opin. Endocrinol. Diabetes Obes. 24, 43–46. 10.1097/MED.0000000000000305 27898587

[B22] Law S. H. Chan M. L. Marathe G. K. Parveen F. Chen C. H. Ke L. Y. (2019). An updated review of lysophosphatidylcholine metabolism in human diseases. Int. J. Mol. Sci. 20:149. 10.3390/ijms20051149 30845751 PMC6429061

[B23] Li F. Li C. Chen Y. Liu J. Zhang C. Irving B.. (2019). Host genetics influence the rumen microbiota and heritable rumen microbial features associate with feed efficiency in cattle. Microbiome 7:92. 10.1186/s40168-019-0699-1 31196178 PMC6567441

[B24] Li-Hawkins J. Gåfvels M. Olin M. Lund E. G. Andersson U. Schuster G.. (2002). Cholic acid mediates negative feedback regulation of bile acid synthesis in mice. J. Clin. Investig. 110, 1191–1200. 10.1172/JCI0216309 12393855 PMC150802

[B25] Meenakumari K. J. Agarwal S. Krishna A. Pandey L. K. (2004). Effects of metformin treatment on luteal phase progesterone concentration in polycystic ovary syndrome. Br. J. Med. Biol. Res. 37, 1637–1644. 10.1590/S0100-879X2004001100007 15517078

[B26] Nagy R. A. Hollema H. Andrei D. Jurdzinski A. Kuipers F. Hoek A.. (2019). The origin of follicular bile acids in the human ovary. Am. J. Pathol. 189, 2036–2045. 10.1016/j.ajpath.2019.06.011 31369754

[B27] National Research Council (1985). Nutrient Requirements of Sheep. Washington, DC: National Research Council.

[B28] Sandovici I. Smith N. H. Nitert M. D. Ackers-Johnson M. Uribe-Lewis S. Ito Y.. (2011). Maternal diet and aging alter the epigenetic control of a promoter-enhancer interaction at the Hnf4a gene in rat pancreatic islets. Proc. Natl. Acad. Sci. U.S.A. 108, 5449–5454. 10.1073/pnas.1019007108 21385945 PMC3069181

[B29] Santos A. Giráldez F. J. Frutos J. Andrés S. (2019). Liver transcriptomic and proteomic profiles of preweaning lambs are modified by milk replacer restriction. J. Dairy Sci. 102, 1194–1204. 10.3168/jds.2018-15110 30594368

[B30] Santos A. Giráldez F. J. Mateo J. Frutos J. Andrés S. (2018b). Programming Merino lambs by early feed restriction reduces growth rates and increases fat accretion during the fattening period with no effect on meat quality traits. Meat Sci. 135, 20–26. 10.1016/j.meatsci.2017.08.007 28843940

[B31] Santos A. Giráldez F. J. Valdés C. Trevisi E. Lucini L. Frutos J.. (2018a). Milk replacer restriction during early life impairs the live body weight and progesterone patterns of ewe lambs during the replacement period. J. Dairy Sci. 101, 8021–8031. 10.3168/jds.2018-14648 29960776

[B32] Santos A. Valdés C. Giráldez F. J. López S. France J. Frutos J.. (2018c). Feed efficiency and the liver proteome of fattening lambs are modified by feed restriction during the suckling period. Animal 12, 1838–1846. 10.1017/S1751731118000046 29362009

[B33] Schulz L. C. (2010). The Dutch Hunger Winter and the developmental origins of health and disease. Proc. Natl. Acad. Sci. U.S.A. 107, 16757–16758. 10.1073/pnas.1012911107 20855592 PMC2947916

[B34] Sinclair K. D. Rutherford K. M. D. Wallace J. M. Brameld J. M. Stöger R. Alberio R.. (2016). Epigenetics and developmental programming of welfare and production traits in farm animals. Reprod. Fertil. Dev. 28, 1443–1478. 10.1071/RD16102 27439952

[B35] Sonne D. P. Hansen M. Knop F. K. (2014). Mechanisms in endocrinology. Bile acid sequestrants in type 2 diabetes: Potential effects on GLP1 secretion. Eur. J. Endocrinol. 171, R47–65. 10.1530/EJE-14-0154 24760535

[B36] Thompson R. P. Nilsson E. Skinner M. K. (2020). Environmental epigenetics and epigenetic inheritance in domestic farm animals. Anim. Reprod. Sci. 220:106316. 10.1016/j.anireprosci.2020.106316 32094003

[B37] van Keulen J. Young B. A. (1977). Evaluation of acid-isoluble ash as a natural marker in ruminant digestibility studies. J. Anim. Sci. 44, 282–287. 10.2527/jas1977.442282x

[B38] Viguié C. Chaillou E. Gayrard V. Picard-Hagen N. Fowler P. A. (2020). Toward a better understanding of the effects of endocrine disrupting compounds on health: human-relevant case studies from sheep models. Mol. Cell. Endocrinol. 505 :110711. 10.1016/j.mce.2020.110711 31954824

[B39] Virant-Klun I. (2015). Postnatal oogenesis in humans: a review of recent findings. Stem Cells Cloning 8, 49–60. 10.2147/SCCAA.S32650 25848307 PMC4376261

[B40] Watanabe M. Houten S. M. Wang L. Moschetta A. Mangelsdorf D. J. Heyman R. A.. (2004). Bile acids lower triglyceride levels via a pathway involving FXR, SHP, and SREBP-1c. J. Clin. Investig. 113, 1408–1418. 10.1172/JCI21025 15146238 PMC406532

[B41] Wreczycka K. Gosdschan A. Yusuf D. Grüning B. Assenov Y. Akalin A. (2017). Strategies for analyzing bisulfite sequencing data. J. Biotechnol. 261, 105–115. 10.1016/j.jbiotec.2017.08.007 28822795

[B42] Wright C. Evans A. C. O. Evans N. P. Duffy P. Fox J. Boland M. P.. (2002). Effect of maternal exposure to the environmental estrogen, octylphenol, during fetal and/or postnatal life on onset of puberty, endocrine status, and ovarian follicular dynamics in ewe lambs. Biol. Reprod. 67, 1734–1740. 10.1095/biolreprod.101.002006 12444047

[B43] Zhang S. Rattanatray L. McMillen I. C. Suter C. M. Morrison J. L. (2011). Periconceptional nutrition and the early programming of a life of obesity or adversity. Prog. Biophys. Mol. Biol. 106, 307–314. 10.1016/j.pbiomolbio.2010.12.004 21168433

