# Supplementary material for: The Role of Feed Restriction on DNA Methylation, Feed Efficiency, Metabolome, Biochemical Profile, and Progesterone Patterns in the Female Filial Generation (F1) Obtained From Early Feed Restricted Ewes (F0)
Source: Front Physiol. 2021 Dec 27;12:779054. doi: 10.3389/fphys.2021.779054 (PMC8745145; doi:10.3389/fphys.2021.779054)

Supplementary Table 1. Ingredients and chemical composition of the commercial lamb milk replacer administered during the artificial rearing period of the lambs

Ingredients, g/kg	
Skim milk powder	500
Milk whey powder	160
Vegetable oil (cocoa)	140
Animal fat (pork lard)	120
Whey protein powder	40
Hydrolyzed wheat protein	15
Yeast/sodium bicarbonate /Dextrose	15
PreVIT mineral[1]	10
Chemical composition, g/kg DM	
DM, g/kg	960
CP	235
Ether extract	260
Ash	70
Metabolizable energy, kcal/kg DM	5089

[1] Provided per kg milk replacer: vitamin A, 80,000 IU; vitamin D_3, 4,250 IU; iron, 40 mg; cobalt, 0.2 mg; copper, 5 mg; manganese, 25 mg; zinc, 30 mg; selenium, 0.2 mg.

Supplementary Table 2. Ingredients and chemical composition of the complete pelleted diet administered to the replacement ewe lambs

Ingredients, g/kg	
Lucerne	360
Barley	145
Corn	140
Barley straw	120
Wheat	70
Wheat bran	65
Soybean meal 44	50
Molasses (sugar cane)	20
Vitamins and minerals	20
Lard	10
Chemical composition, g/kg DM	
DM, g/kg	904
aNDF[1]	306
ADF	180
CP	139
Ash	90.6

[1] Amylase-treated neutral detergent fiber.

Supplementary Table 3. Total enriched GO terms from the list of differentially methylated genes in the female progeny (F1-RES) of early feed restricted ewes.

Functional Category	Enrichment FDR	Genes in list	Total genes	Genes symbol
Actin cytoskeleton organization	0.0349	17	687	*MYOM2 MYOM1* ***MYH14*** *APOA1 SORBS3 TSC1 PRKN NOX4 DPYSL3 PREX1 BCAS3 ABLIM2* ***GPM6B CGNL1*** *MYO18A CYFIP1* ***TENM1***
Actin filament organization	0.0437	12	426	*MYOM2 MYOM1 APOA1 SORBS3 TSC1 PRKN NOX4 DPYSL3 PREX1* ***CGNL1*** *CYFIP1* ***TENM1***
Actin filament-based process	0.0284	19	779	*MYOM2 MYOM1 KCNE5* ***MYH14*** *APOA1 SORBS3 TSC1 PRKN NOX4 DPYSL3 PREX1 BCAS3 ABLIM2* ***GPM6B CGNL1*** *RNF207 MYO18A CYFIP1* ***TENM1***
Action potential	0.0446	6	131	***CACNA1I*** *KCNE5 P2RX1* ***MYH14*** *RNF207* ***FGF13***
Actomyosin structure organization	0.0315	8	198	*MYOM2 MYOM1* ***MYH14*** *APOA1 SORBS3 TSC1 NOX4 MYO18A*
Adult locomotory behavior	0.0104	6	83	*AGTPBP1 APP* ***TSHR*** *TSC1 PRKN FOXA2*
Amide transport	0.0222	42	2265	***SEC31B*** *TNPO1 SCAMP1* ***NLRC4 NUP188*** *TNFRSF21* ***GATA3*** *CRTAM APOA1 PPARG* ***STXBP4*** *PRKN* ***NDUFA13*** *INSIG1* ***GPM6B*** *KLHL20* ***ATG16L1 HNF4A*** *AAGAB* ***MAPK10 SIL1*** *BCAS3* ***LYST UEVLD*** <u>*ADCY8*</u> *AGAP1 ITSN1 DENND1B MYOM1* ***LMAN2L MCU*** *TSC1* ***NPLOC4*** *MYO18A* ***TENM1*** *FOXA2* ***HERC2*** *ACAA1 ATP6V0D2* ***ITPR1 PRKCB*** *KIF13B*
Anatomical structure morphogenesis	0.0077	52	2785	*MYOM2* ***EFNB1*** *MYOM1* ***FAM20A*** *FOXA2* ***KLK8 CDH13*** <u>*COL6A3*</u> ***PTK2*** *CYFIP1 APOA1* ***UNC13A*** *PPARG ADAMTS12 ADGRB1 KIF13B* <u>*TRAF3IP1*</u> *NEO1 CRMP1 NOX4* ***TBX5 HNF4A MYH14 GATA3*** *BVES* ***MDFI*** *ACVR2A PREX1 CITED1 AGTPBP1 BCAS3 APP GNA12* ***DPYSL4*** *RDH13 DISC1* ***NFASC TSHR DRGX*** *TSC1* ***PRKCB*** *PRKN RXRA INSIG1 NTRK1* ***MYO9A*** *EPN2 DNMBP* ***FGF13*** *RNF207* ***ATP7A RANBP9***
Aspartate family amino acid biosynthetic process	0.0303	3	22	*PLOD2 ADI1 MTR*
Axon development	0.0013	19	517	***EFNB1*** *CYFIP1 KIF13B NEO1 CRMP1* ***GATA3*** *MTR APOA1* ***KLK8*** *APP TNFRSF21 DISC1* ***NFASC DRGX PTK2*** *NTRK1* ***FGF13 RANBP9*** *ADGRB1*
Axon guidance	0.0095	11	274	***EFNB1*** *CYFIP1 NEO1 CRMP1* ***GATA3*** *APP* ***NFASC DRGX*** *NTRK1* ***RANBP9 PTK2***

Axonogenesis	0.0105	15	474	**EFNB1** CYFIP1 KIF13B NEO1 CRMP1 **GATA3** APP DISC1 **NFASC DRGX PTK2** NTRK1 **FGF13 RANBP9** ADGRB1
Behavior	0.0349	16	627	**KLK8** HDAC4 **BRINP1** AGTPBP1 APP ADCY8 **TSHR** TSC1 GRID1 PRKN NTRK1 **MYH14** FOXA2 **FGF13 ZFHX3 ATP7A**
Biological adhesion	0.0070	34	1548	**TENM1 CDH13** TNFRSF21 COL26A1 **PTK2** FOXA2 **CDK6** BVES APOA1 SKAP1 TSC1 CD99 NEO1 **KIFAP3 PTPRC EFNB1** RAPGEF1 **GATA3** SORBS3 PREX1 TRIM29 ITGA11 BCAS3 APP **ITGA9** HMCN2 DISC1 COL6A3 **NFASC** PEAR1 **GPM6B** ADAMTS12 **ZFHX3** ADGRB1
Cardiac muscle cell differentiation	0.0117	7	119	MYOM2 MYOM1 NOX4 **TBX5** BVES TSC1 RXRA
Cardiocyte differentiation	0.0284	7	150	MYOM2 MYOM1 **TBX5** NOX4 BVES TSC1 RXRA
CD4-positive. alpha-beta T cell cytokine production	0.0303	3	22	DENND1B IL18R1 **GATA3**
Cell activation	0.0354	31	1591	TNFRSF21 **LAT MLH1 PTPRC EFNB1 CDK6 GATA3** P2RX1 IL18R1 PREX1 **TSHR** TSC1 **PRKCB** INPP5D PEAR1 NTRK1 APP MYO18A **ATP7A** KIF13B ACAA1 HDAC4 SCAMP1 FRK **C1orf35** GNA12 **ITPR1 NFASC** CTSB **DYNC1H1** CYFIP1
Cell adhesion	0.0068	34	1541	**TENM1 CDH13** TNFRSF21 COL26A1 **PTK2** FOXA2 **CDK6** BVES APOA1 SKAP1 TSC1 CD99 NEO1 **KIFAP3 PTPRC EFNB1** RAPGEF1 **GATA3** SORBS3 PREX1 TRIM29 ITGA11 BCAS3 APP **ITGA9** HMCN2 DISC1 COL6A3 **NFASC** PEAR1 **GPM6B** ADAMTS12 **ZFHX3** ADGRB1
Cell development	0.0011	49	2230	**TENM1** MYOM2 **GPM6B BRINP1 EFNB1** MYOM1 **KLK8 ZFHX3** COL6A3 NTRK1 CYFIP1 **CDK6** APOA1 **UNC13A** PPARG KIF13B NEO1 HDAC4 CRMP1 **MLH1 HNF4A** RAPGEF1 **GATA3 NEURL1** BVES DPYSL3 MTR PREX1 **CDK5RAP2** APP TNFRSF21 **DPYSL4** RDH13 DISC1 **NFASC TSHR DRGX** TSC1 **PTK2 TOPAZ1** PRKN CATSPER4 ZNF536 ITSN1 **PTPRC FGF13 ATP7A RANBP9** ADGRB1
Cell differentiation	0.0002	84	4459	ITGA11 SND1 **TENM1** MYOM2 **GPM6B BRINP1 EFNB1** MYOM1 **GATA3** FRK BVES FOXA2 CITED1 **KLK8** PPARG **ZFHX3** COL6A3 DEUP1 **PTK2** NR1D2 ETV4 NTRK1 CYFIP1 KAT6A **TBX5 CDK6** APOA1 ACVR2A **UNC13A** ADAMTS7 ADAMTS12 KIF13B NEO1 HDAC4 CRMP1 **MLH1 PTPRC** NOX4 **HNF4A** SLC7A5 USP42 RAPGEF1 **NEURL1 MDFI** DPYSL3 IL18R1 MTR

				PREX1 AGTPBP1 **CDK5RAP2** UNC45A APP TNFRSF21 GNA12 **DPYSL4** TDRD9 **CTBP1** RDH13 DISC1 **NFASC TSHR DRGX** TSC1 INPP5D **TOPAZ1** ADGRB1 PRKN RXRA INSIG1 INSC CATSPER4 **TXNRD3** ZNF536 **GRK5** ITSN1 ACADVL CTSB **MYO9A LDLRAD4 FGF13 MDGA2 ATP7A RANBP9 PRKCB**
Cell migration	0.0212	31	1506	**TBX5 GATA3** PPARG **CDH13 PTK2** ADGRB1 HDAC4 NOX4 **EFNB1** BVES DPYSL3 APOA1 PREX1 BCAS3 **ITGA9** GNA12 DISC1 **ATP7A DRGX MAP2K3 PTPRC** ITGA11 SKAP1 APP ADAMTS12 **LDLRAD4** MYO18A **FGF13 LYST** SLC7A5 INPP5D
Cell morphogenesis	0.0017	29	1067	**EFNB1 KLK8 CDH13** COL6A3 CYFIP1 APOA1 **UNC13A** KIF13B NEO1 CRMP1 NOX4 **MYH14 GATA3** BVES PREX1 APP GNA12 **DPYSL4** DISC1 **NFASC DRGX PTK2** PRKN NTRK1 DNMBP **FGF13 ATP7A RANBP9** ADGRB1
Cell morphogenesis involved in differentiation	0.0222	19	756	**EFNB1** COL6A3 CYFIP1 APOA1 KIF13B NEO1 CRMP1 **GATA3** BVES PREX1 APP DISC1 **NFASC DRGX PTK2** NTRK1 **FGF13 RANBP9** ADGRB1
Cell morphogenesis involved in neuron differentiation	0.0437	15	598	**EFNB1** CYFIP1 KIF13B NEO1 CRMP1 **GATA3** APP DISC1 **NFASC DRGX PTK2** NTRK1 **FGF13 RANBP9** ADGRB1
Cell motility	0.0068	36	1670	**TBX5 GATA3** PPARG **CDH13 PTK2** ADGRB1 HDAC4 NOX4 **EFNB1 CACNA1I NEURL1** BVES DPYSL3 APOA1 PREX1 BCAS3 **ITGA9** GNA12 DISC1 **ATP7A DRGX** DPCD CATSPER4 **MAP2K3 PTPRC** ITGA11 SKAP1 APP ADAMTS12 **LDLRAD4** MYO18A **CDK6 FGF13 LYST** SLC7A5 INPP5D
Cell part morphogenesis	0.0037	21	701	**EFNB1 KLK8** CYFIP1 **UNC13A** KIF13B NEO1 CRMP1 **GATA3** APP **DPYSL4** DISC1 **NFASC DRGX PTK2** PRKN NTRK1 **MYH14 FGF13 ATP7A RANBP9** ADGRB1
Cell projection assembly	0.0437	15	597	ABLIM2 CYFIP1 **CDH13** HDAC4 **NEURL1** DPYSL3 BCAS3 DISC1 **ATP7A** TRAF3IP1 **TENM1 KIFAP3** TNPO1 **CDK5RAP2 DYNC1H1**
Cell projection morphogenesis	0.0052	20	682	**EFNB1 KLK8** CYFIP1 **UNC13A** KIF13B NEO1 CRMP1 **GATA3** APP **DPYSL4** DISC1 **NFASC DRGX PTK2** PRKN NTRK1 **FGF13 ATP7A RANBP9** ADGRB1
Cell projection organization	0.0001	43	1589	**GPM6B EFNB1 KLK8** ABLIM2 NTRK1 TRAF3IP1 CYFIP1 **UNC13A CDH13** KIF13B DNAH9 NEO1 HDAC4 CRMP1 RAPGEF1 **GATA3 NEURL1** DPYSL3

				MTR APOA1 PREX1 BCAS3 APP TNFRSF21 ***DPYSL4*** *DISC1* ***NFASC ATP7A*** *DEUP1* ***TSHR DRGX*** *TSC1* ***PTK2*** *PRKN ITSN1* ***TENM1 FGF13 RANBP9 KIFAP3*** *TNPO1* ***CDK5RAP2*** *ADGRB1* ***DYNC1H1***
Cell proliferation	0.0178	41	2165	*TNFRSF21* ***PTK2 TBX5 GATA3 NEURL1*** *PPARG* ***CDH13*** <u>*GAREM1*</u> *APP NTRK1* <u>*HDAC4*</u> ***KIFAP3 PTPRC*** *NOX4* ***EFNB1 CDK6*** <u>*RAPGEF1*</u> *APOA1 ACVR2A* ***KLK8 PEMT*** *RPTOR DISC1* ***ATP7A*** *TSC1 INPP5D* ***ARNT2*** *RXRA* ***HNF4A PBRM1 STXBP4 GRK5 FGF13 IFNLR1 TENM1*** *FRK CITED1* ***CTBP1*** *MST1R* ***TSHR*** *ADGRB1*
Cell surface receptor signaling pathway	0.0077	59	3287	***EFNB1 NEURL1*** *FRK ACVR2A MST1R OTUD4 TSC1* ***CSNK1G1 PTK2 IFNLR1*** *PEAR1 NTRK1 DENND1B AXIN1 APP* ***PTPRC*** <u>*RAPGEF1*</u> *IL18R1 APOA1 CITED1* <u>*GAREM1*</u> *ADAMTS12* ***GRIK3 LDLRAD4 RUVBL1*** *PRKN* ***LAT*** *NEO1* ***HNF4A CDK6 GATA3*** *P2RX1* ***UNC13A*** *ITGA11* ***ITGA9*** *TNFRSF21 DISC1* ***STXBP4 PRKCB*** *ADGRB1 GRID1 SIGIRR* ***NDUFA13 IL17REL*** *ADAMTSL2* ***GRK5*** <u>*TRAF3IP1*</u> *CYFIP1 EPN2 PPARG* ***DSTYK CDH13*** *SKAP1* ***TSHR MDFI MAPK10*** *ATP6V0D2 INPP5D ITSN1*
Cell-cell signaling	0.0084	37	1774	***UNC13A*** *SYN2* ***GRIK3*** *MST1R HTR1E* ***CSNK1G1*** *GRID1 AXIN1 APP* ***TBX5 STXBP4 RUVBL1*** *PRKN* ***HNF4A*** <u>*RAPGEF1*</u> ***GATA3 NEURL1*** *P2RX1* <u>*ADCY8*</u> *DISC1* ***PRKCB*** *ADGRB1 NTRK1* ***GRK5*** *ITSN1 CYFIP1* ***MCU*** *KCNE5* ***MDFI*** *FOXA2 RNF207* ***EFNB1*** *GPR50 CITED1* ***FGF13 ITPR1 TSHR***
Cell-matrix adhesion	0.0117	10	239	***CDK6*** *TSC1 BCAS3 DISC1 SKAP1* ***PTK2 GPM6B*** *ITGA11* ***CDH13*** *ADAMTS12*
Cell-substrate adhesion	0.0018	15	358	<u>*COL26A1*</u> ***CDK6*** *APOA1 TSC1 BVES SORBS3 PREX1 BCAS3 DISC1 SKAP1* ***PTK2 GPM6B*** *ITGA11* ***CDH13*** *ADAMTS12*
Cellular component assembly	0.0084	59	3309	*APP MYOM2* ***SEC31B ATG16L1 NLRC4*** *MYOM1* ***EIF4H*** *ATG2A APOA1* ***UNC13A*** *ERAL1* ***CDH13 ATG12*** *NDUFAF6 ABLIM2 DEUP1* ***RUVBL1 BRF1*** *CYFIP1 AXIN1 SORBS3 SKAP1* ***MCU*** *TSC1* ***LDLRAD4*** *TRAPPC12 PRKN RXRA* <u>*HDAC4*</u> ***MLH1*** *KAT6A NOX4* ***NEURL1*** *P2RX1 DPYSL3 PREX1* ***CDK5RAP2*** *BCAS3 DISC1* ***NFASC ATP7A PTK2*** *ADGRB1* ***DYNC1H1*** *NTRK1* <u>*TRAF3IP1*</u> ***GPM6B RNF4 MYO9A*** <u>*RAPGEF1*</u> ***NDUFA13*** *INSIG1* ***TENM1 FGF13*** <u>*ADCY8*</u> *ACACA* ***RANBP9 KIFAP3*** *TNPO1*
Cellular component biogenesis	0.0284	59	3556	*APP MYOM2* ***SEC31B ATG16L1 NLRC4*** *MYOM1* ***EIF4H*** *ATG2A APOA1* ***UNC13A*** *ERAL1* ***CDH13 ATG12*** *NDUFAF6 ABLIM2 DEUP1* ***RUVBL1 BRF1***

				CYFIP1 AXIN1 SORBS3 SKAP1 **MCU** TSC1 **LDLRAD4** TRAPPC12 PRKN RXRA HDAC4 **MLH1** KAT6A NOX4 **NEURL1** P2RX1 DPYSL3 PREX1 **CDK5RAP2** BCAS3 DISC1 **NFASC** **ATP7A** **PTK2** ADGRB1 **DYNC1H1** NTRK1 TRAF3IP1 **GPM6B** **RNF4** **MYO9A** RAPGEF1 **NDUFA13** INSIG1 **TENM1** **FGF13** ADCY8 ACACA **RANBP9** **KIFAP3** TNPO1
Cellular component morphogenesis	0.0016	31	1172	MYOM2 **EFNB1** MYOM1 **KLK8** **CDH13** COL6A3 CYFIP1 APOA1 **UNC13A** KIF13B NEO1 CRMP1 NOX4 **MYH14** **GATA3** BVES PREX1 APP GNA12 **DPYSL4** DISC1 **NFASC** **DRGX** **PTK2** PRKN NTRK1 DNMBP **FGF13** **ATP7A** **RANBP9** ADGRB1
Cellular developmental process	0.0002	87	4671	ITGA11 SND1 **TENM1** MYOM2 **GPM6B** **BRINP1** **EFNB1** MYOM1 **GATA3** FRK BVES FOXA2 CITED1 **KLK8** PPARG **ZFHX3** **CDH13** COL6A3 DEUP1 **PTK2** NR1D2 ETV4 NTRK1 CYFIP1 KAT6A **TBX5** **CDK6** APOA1 ACVR2A **UNC13A** ADAMTS7 ADAMTS12 KIF13B NEO1 HDAC4 CRMP1 **MLH1** **PTPRC** NOX4 **HNF4A** SLC7A5 **MYH14** USP42 RAPGEF1 **NEURL1** **MDFI** DPYSL3 IL18R1 MTR PREX1 AGTPBP1 **CDK5RAP2** UNC45A APP TNFRSF21 GNA12 **DPYSL4** TDRD9 **CTBP1** RDH13 DISC1 **NFASC** **TSHR** **DRGX** TSC1 INPP5D **TOPAZ1** ADGRB1 PRKN RXRA INSIG1 INSC CATSPER4 **TXNRD3** ZNF536 **GRK5** ITSN1 ACADVL CTSB **MYO9A** DNMBP **LDLRAD4** **FGF13** **MDGA2** **ATP7A** **RANBP9** **PRKCB**
Cellular localization	0.0083	56	3087	**CLEC16A** **GPM6B** **SEC31B** KLHL20 TNPO1 **NUP188** **LMAN2L** **UNC13A** **ITPR1** SYN2 **DHRS7C** **DYNC1H1** TRAF3IP1 DENND1B PRKN EIPR1 CITED1 MAU2 **STXBP4** **NDUFA13** INSIG1 **MLH1** **PTPRC** **ATG16L1** P2RX1 **MAPK10** BVES **CDK5RAP2** TRIM29 SKAP1 BCAS3 APP ADCY8 DISC1 **NFASC** **PRKCB** **PTK2** ITSN1 **LAT** CYFIP1 **RRAGB** **CDH13** **LYST** TSC1 TRAPPC12 **NPLOC4** **TENM1** FOXA2 **FGF13** **SIL1** **HERC2** ACAA1 **KIFAP3** PPP1R13B KIF13B ACACA
Cellular protein modification process	0.0411	69	4434	EIPR1 **MAP2K3** KLHL20 KAT6A **CDK6** USP42 **MAPK10** **SUDS3** FRK PPP2R5D LOXL1 RPTOR **ATG12** PLOD2 OTUD4 **STK32C** **PRKCB** **CSNK1G1** **PTK2** **RUVBL1** ALG12 PRKN NTRK1 **LAT** HDAC4 **PTPRC** RCOR1 AXIN1 **FAM20A** APOA1 KDM4B **HERC2** **FGF13** APP MST1R SUPT3H **GRK5** **RNF4** **ATG16L1** NOX4 RAPGEF1 **GATA3** **NEURL1** PTPN5 **PRKD3** SORBS3

				RNF170 ACVR2A **LRRC29** UBAC1 **DSTYK** AGTPBP1 **UEVLD** TSC1 ADGRB1 ZNRF1 **FUT4** **RANBP9** GAREM1 **CTBP1** **LDLRAD4** TRAF3IP1 **TENM1** ADCY8 **ATP7A** AGBL1 **NUP188** **FBXL16** WDR20
Cellular response to chemical stimulus	0.0034	65	3536	ACAA1 **BRINP1** **RRAGB** **MAPK10** ACVR2A PPARG RPTOR OTUD4 **TSHR** TSC1 NR1D2 PRKN **IFNLR1** NTRK1 RAPGEF1 IL18R1 APOA1 CITED1 **DSTYK** ADAMTS7 BCAS3 GAREM1 TNFRSF21 ADAMTS12 **STXBP4** **PTK2** **NDUFA13** INSIG1 NEO1 HDAC4 **PTPRC** NOX4 **HNF4A** **GATA3** **NEURL1** MTR PREX1 APP **ITGA9** ADCY8 MST1R **ATP7A** **PRKCB** **LDLRAD4** SIGIRR RXRA **IL17REL** **TXNRD3** ADAMTSL2 CYFIP1 CTSB **SERPINB9** **MAP2K3** EPN2 SKAP1 DPYSL3 FOXA2 **LYST** **RNF4** ACADVL AXIN1 PTPN5 ATP6V0D2 INPP5D **ARNT2**
Cellular response to endogenous stimulus	0.0012	36	1432	**RRAGB** ACVR2A PPARG RPTOR **TSHR** TSC1 NR1D2 NTRK1 RAPGEF1 CITED1 **DSTYK** ADAMTS7 GAREM1 ADAMTS12 **PTK2** NEO1 HDAC4 NOX4 **HNF4A** **GATA3** **NEURL1** APP ADCY8 **ATP7A** **STXBP4** **LDLRAD4** RXRA ADAMTSL2 CYFIP1 CTSB SKAP1 **PRKCB** **RNF4** AXIN1 ATP6V0D2 PRKN
Cellular response to growth factor stimulus	0.0034	21	694	ACVR2A NTRK1 RAPGEF1 CITED1 **DSTYK** ADAMTS7 GAREM1 ADAMTS12 **PTK2** NEO1 NOX4 **GATA3** APP **ATP7A** **PRKCB** **LDLRAD4** ADAMTSL2 CYFIP1 **MAP2K3** EPN2 SKAP1
Cellular response to nitrogen compound	0.0168	18	675	**RRAGB** PPARG RPTOR TSC1 RAPGEF1 **PTK2** NOX4 **NEURL1** MTR APP ADCY8 **ATP7A** **STXBP4** CYFIP1 **TSHR** **PRKCB** ATP6V0D2 PRKN
Cellular response to organic substance	0.0041	56	2938	**BRINP1** **RRAGB** **MAPK10** ACVR2A PPARG RPTOR OTUD4 **TSHR** TSC1 NR1D2 **IFNLR1** NTRK1 RAPGEF1 IL18R1 APOA1 CITED1 **DSTYK** ADAMTS7 GAREM1 TNFRSF21 ADAMTS12 **STXBP4** **PTK2** PRKN **NDUFA13** INSIG1 NEO1 HDAC4 **PTPRC** NOX4 **HNF4A** **GATA3** **NEURL1** APP ADCY8 MST1R **ATP7A** **PRKCB** **LDLRAD4** SIGIRR RXRA **IL17REL** ADAMTSL2 CYFIP1 CTSB **MAP2K3** EPN2 SKAP1 DPYSL3 FOXA2 **RNF4** ACADVL AXIN1 PTPN5 ATP6V0D2 INPP5D
Cellular response to organonitrogen compound	0.0137	17	597	**RRAGB** PPARG RPTOR TSC1 RAPGEF1 **PTK2** NOX4 **NEURL1** APP ADCY8 **ATP7A** **STXBP4** CYFIP1 **TSHR** **PRKCB** ATP6V0D2 PRKN

Chemical homeostasis	0.0409	25	1212	*APOA1 SLC12A9 ATP6V0D2* ***ITPR1 MCU*** *SLC9C2* ***ATP7A*** *PNPLA1* ***DHRS7C STXBP4*** *INSIG1 NEO1* ***PTPRC*** *NOX4* ***HNF4A*** *P2RX1 APP ADCY8 DISC1* ***PRKCB*** *NR1D2* ***FAM20A*** *PPARG FOXA2 PRKN*
Chemotaxis	0.0335	17	680	***EFNB1*** *CYFIP1 APOA1* ***CDH13*** *NEO1 CRMP1* ***GATA3*** *PREX1 APP* ***ITGA9 DPYSL4 NFASC DRGX*** *NTRK1* ***LYST RANBP9 PTK2***
C-terminal protein amino acid modification	0.0019	4	15	***ATG12 ATG16L1*** *AGTPBP1 AGBL1*
C-terminal protein deglutamylation	0.0162	2	4	*AGTPBP1 AGBL1*
C-terminal protein lipidation	0.0354	2	7	***ATG12 ATG16L1***
Cytokine biosynthetic process	0.0483	6	134	***MAP2K3 PTPRC GATA3*** *INPP5D SIGIRR APP*
Cytokine metabolic process	0.0494	6	135	***MAP2K3 PTPRC GATA3*** *INPP5D SIGIRR APP*
Cytoskeleton organization	0.0084	31	1386	*MYOM2 MYOM1 DEUP1* ***DYNC1H1 MYH14*** *APOA1 SORBS3* ***CDK5RAP2*** *TSC1 PRKN CRMP1* ***MLH1*** *NOX4* ***NEURL1*** *DPYSL3 PREX1 BCAS3 ABLIM2* ***PTK2*** *TRAF3IP1* ***GPM6B RNF4 CGNL1*** *SKAP1 DISC1 MYO18A CYFIP1* ***TENM1 FGF13 RANBP9*** *KLHL20*
Endothelial cell migration	0.0354	8	206	*PPARG* ***CDH13*** *ADGRB1 APOA1 BCAS3* ***PTK2 MAP2K3 GATA3***
Enzyme linked receptor protein signaling pathway	0.0083	26	1072	***EFNB1*** *FRK ACVR2A MST1R TSC1* ***PTK2*** *NTRK1 CITED1 GAREM1 ADAMTS12* ***LDLRAD4*** *NEO1* ***HNF4A*** *RAPGEF1* ***GATA3 NEURL1*** *APP* ***STXBP4 PRKCB*** *ADAMTSL2 CYFIP1 EPN2* ***DSTYK CDH13*** *ATP6V0D2 ITSN1*
Establishment of cell polarity	0.0178	7	134	***GATA3 CDK5RAP2*** *BCAS3* ***MYO9A*** *MYO18A* ***FGF13 PTK2***
Establishment of localization in cell	0.0051	47	2364	***CLEC16A GPM6B SEC31B*** *KLHL20 TNPO1* ***NUP188 LMAN2L UNC13A ITPR1*** *SYN2* ***DHRS7C DYNC1H1*** *TRAF3IP1 DENND1B EIPR1 CITED1* ***STXBP4 NDUFA13*** *INSIG1* ***MLH1 PTPRC*** *P2RX1* ***MAPK10*** *BVES* ***CDK5RAP2*** *BCAS3 APP ADCY8* ***PRKCB PTK2*** *PRKN ITSN1* ***LAT*** *CYFIP1* ***LYST*** *TSC1 TRAPPC12* ***NPLOC4 TENM1*** *FOXA2* ***SIL1 HERC2*** *ACAA1* ***KIFAP3*** *PPP1R13B KIF13B ACACA*
Establishment of protein localization	0.0180	43	2308	***SEC31B*** *TNPO1 SCAMP1* ***NLRC4 NUP188*** *TNFRSF21* ***GATA3*** *CRTAM APOA1 PPARG* ***STXBP4*** *PRKN* ***NDUFA13*** *INSIG1* ***GPM6B*** *KLHL20* ***ATG16L1 HNF4A*** *AAGAB* ***MAPK10 SIL1*** *BCAS3* ***LYST UEVLD*** *ADCY8 AGAP1 ITSN1 DENND1B MYOM1* ***LMAN2L MCU*** *TSC1* ***NPLOC4*** *MYO18A*

				TENM1 *FOXA2* ***HERC2*** *ACAA1* *PPP1R13B* *ATP6V0D2* ***ITPR1*** ***PRKCB*** *KIF13B*
Extraocular skeletal muscle development	0.0162	2	4	*MYOM2 MYOM1*
Generation of neurons	0.0002	42	1575	***TENM1*** ***GPM6B*** ***BRINP1*** ***EFNB1*** ***KLK8*** ***ZFHX3*** *NTRK1* *CYFIP1* ***UNC13A*** *KIF13B* *NEO1* *CRMP1* <u>*RAPGEF1*</u> ***GATA3*** ***NEURL1*** *DPYSL3* *MTR* *APOA1* *PREX1* *PPARG* *AGTPBP1* ***CDK5RAP2*** *APP* *TNFRSF21* ***DPYSL4*** *RDH13* *DISC1* ***NFASC*** ***TSHR*** ***DRGX*** *TSC1* ***PTK2*** *PRKN* *ZNF536* *ITSN1* *FOXA2* ***CDK6*** ***FGF13*** ***MDGA2*** ***ATP7A*** ***RANBP9*** *ADGRB1*
Homeostatic process	0.0485	36	2004	*APOA1* *SLC12A9* *ATP6V0D2* ***ITPR1*** ***MCU*** *SLC9C2* ***ATP7A*** *PNPLA1* ***DHRS7C*** ***TXNRD3*** *ACVR2A* ***STXBP4*** *INSIG1* *NEO1* *ACADVL* ***PTPRC*** *NOX4* ***HNF4A*** ***GATA3*** *P2RX1* *IL18R1* ***PEMT*** *APP* <u>*ADCY8*</u> *DISC1* ***TSHR*** ***PRKCB*** *INPP5D* *NR1D2* ***DYNC1H1*** ***LAT*** ***CDK6*** ***FAM20A*** *PPARG* *PRKN* *FOXA2*
Immune effector process	0.0147	30	1392	*DENND1B* *CRTAM* *IL18R1* *APOA1* *SUSD4* ***IFNLR1*** ***MLH1*** ***PTPRC*** <u>*RAPGEF1*</u> ***GATA3*** *TSC1* *INPP5D* <u>*TRAF3IP1*</u> ***LAT*** ***SERPINB9*** *APP* ***LYST*** ***NPLOC4*** *MYO18A* ***ATP7A*** *ACAA1* *SCAMP1* *P2RX1* *FRK* ***C1orf35*** ***NFASC*** *CTSB* ***PTK2*** ***DYNC1H1*** *CYFIP1*
Immune system process	0.0349	58	3539	***MLH1*** ***NLRC4*** ***GATA3*** *TNFRSF21* *OTUD4* *DENND1B* ***LAT*** ***PTPRC*** *KAT6A* ***CDK6*** *CRTAM* *IL18R1* *APOA1* *ACVR2A* *PPARG* *SKAP1* *SUSD4* ***SERPINB9*** ***IFNLR1*** ***EFNB1*** <u>*RAPGEF1*</u> *BVES* *PREX1* *CITED1* *TRIM29* ***ITGA9*** *MST1R* ***TSHR*** *TSC1* ***STXBP4*** ***PRKCB*** *INPP5D* *DEFB123* *ADGRB1* *NTRK1* <u>*TRAF3IP1*</u> *APP* ***LYST*** ***NPLOC4*** *MYO18A* ***ATP7A*** *KIF13B* *CD99* ***TENM1*** *ACAA1* <u>*HDAC4*</u> ***KIFAP3*** *SCAMP1* *SLC7A5* *P2RX1* ***MAPK10*** *FRK* ***C1orf35*** ***NFASC*** *CTSB* ***PTK2*** ***DYNC1H1*** *CYFIP1*
Interleukin-5 secretion	0.0411	2	8	*TNFRSF21* ***GATA3***
Intracellular signal transduction	0.0138	55	3113	***MAP2K3*** ***RRAGB*** ***MAPK10*** ***PRKD3*** *RPTOR* *GNA12* <u>*ADCY8*</u> *MST1R* ***TSHR*** *TSC1* ***STK32C*** ***PRKCB*** *ADGRB1* *NTRK1* ***LAT*** *PPP1R13B* *APOA1* ***FGF13*** ***CDH13*** ***ITPR1*** ***WWC2*** ***MCU*** *PRKN* ***MYO9A*** <u>*HDAC4*</u> *LMCD1* ***MLH1*** ***PTPRC*** *NOX4* <u>*RAPGEF1*</u> ***GATA3*** *DNMBP* *IL18R1* *SORBS3* *PREX1* ***UNC13A*** *DISC1* *INPP5D* *PEAR1* *ITSN1* *APP* ***RANBP9*** ***CLEC16A*** *MYOM1* ***CGNL1*** ***DSTYK*** <u>*GAREM1*</u> ***PTK2*** ***NPLOC4*** ***NDUFA13*** *CYFIP1* ***TENM1*** *AXIN1* *NKIRAS1* *KAT6A*

Ion transport	0.0437	33	1775	***GPM6B CACNA1I SLC25A14 CLCN7*** *P2RX1 APOA1 SLC12A9* ***ITPR1 MCU*** *SLC9C2* ***ATP7A*** *KCNE5* ***DHRS7C PTPRC*** *SLC7A5 SLC22A18 SLC16A10 SLC26A10 SLC41A2 ATP6V0D2 SLC5A10* ***GRIK3*** *TSC1* ***PRKCB*** *GRID1 PRKN CATSPER4 RNF207* ***FGF13*** *PPARG APP RXRA ACACA*
Ionotropic glutamate receptor signaling pathway	0.0373	3	25	*APP* ***GRIK3*** *GRID1*
Lamellipodium assembly	0.0178	5	65	*ABLIM2 CYFIP1* ***CDH13*** *HDAC4* ***ATP7A***
Lamellipodium organization	0.0354	5	83	*ABLIM2 CYFIP1* ***CDH13*** *HDAC4* ***ATP7A***
Leukocyte activation	0.0411	28	1416	*TNFRSF21* ***LAT MLH1 PTPRC EFNB1 CDK6 GATA3*** *IL18R1 PREX1* ***TSHR*** *TSC1* ***PRKCB*** *INPP5D NTRK1 APP MYO18A* ***ATP7A*** *KIF13B ACAA1 HDAC4 SCAMP1 P2RX1 FRK* ***C1orf35 NFASC*** *CTSB* ***DYNC1H1*** *CYFIP1*
Localization of cell	0.0068	36	1670	***TBX5 GATA3*** *PPARG* ***CDH13 PTK2*** *ADGRB1 HDAC4 NOX4* ***EFNB1 CACNA1I NEURL1*** *BVES DPYSL3 APOA1 PREX1 BCAS3* ***ITGA9*** *GNA12 DISC1* ***ATP7A DRGX*** *DPCD CATSPER4* ***MAP2K3 PTPRC*** *ITGA11 SKAP1 APP ADAMTS12* ***LDLRAD4*** *MYO18A* ***CDK6 FGF13 LYST*** *SLC7A5 INPP5D*
Locomotion	0.0012	44	1921	***EFNB1*** *CYFIP1* ***TBX5 GATA3*** *APOA1 PPARG* ***CDH13 PTK2*** *ADGRB1 NEO1 HDAC4 CRMP1 NOX4* ***CACNA1I NEURL1*** *BVES DPYSL3 PREX1 BCAS3 APP* ***ITGA9*** *GNA12* ***DPYSL4*** *DISC1* ***NFASC ATP7A TSHR DRGX*** *DPCD CATSPER4 NTRK1* ***MAP2K3 PTPRC*** *ITGA11 SKAP1 ADAMTS12* ***LDLRAD4*** *MYO18A* ***CDK6 FGF13 LYST RANBP9*** *SLC7A5 INPP5D*
Locomotory behavior	0.0149	9	207	*AGTPBP1 APP ADCY8* ***TSHR*** *TSC1 PRKN FOXA2* ***ZFHX3 ATP7A***
Lymphocyte differentiation	0.0418	11	368	***PTPRC CDK6 GATA3*** *IL18R1 PREX1* ***TSHR*** *TSC1 INPP5D NTRK1* ***ATP7A*** *HDAC4*
Macromolecule localization	0.0307	56	3354	***RUVBL1 SEC31B*** *TNPO1 SCAMP1* ***NLRC4 NUP188*** *APOA1 PPARG TNFRSF21 PRKN* ***GATA3*** *CRTAM MAU2* ***STXBP4 NDUFA13*** *INSIG1* ***GPM6B KIFAP3*** *KLHL20* ***ATG16L1 HNF4A*** *AAGAB* ***MAPK10 SIL1*** *TRIM29 SKAP1 BCAS3* ***LYST UEVLD*** *ADCY8 AGAP1 DISC1* ***NFASC*** *ITSN1 DENND1B MYOM1* ***RRAGB LMAN2L MCU*** *TSC1 TRAPPC12* ***NPLOC4*** *MYO18A* ***TENM1*** *FOXA2* ***FGF13 HERC2*** *ACAA1 PPP1R13B* ***MDFI*** *ATP6V0D2* ***ITPR1 PRKCB*** *RXRA KIF13B ACACA*
Mating	0.0330	4	48	*HDAC4 P2RX1 ACVR2A APP*

Microtubule-based process	0.0166	20	789	*DNAH9 DEUP1* ***DYNC1H1*** *KIF13B* <u>*TRAF3IP1*</u> ***CDK5RAP2*** *CRMP1* ***KIFAP3 MLH1 NEURL1*** *BCAS3 APP DPCD* ***PTK2 RNF4*** *SKAP1 DISC1* ***FGF13 RANBP9*** *TNPO1*
Modulation of chemical synaptic transmission	0.0387	13	467	***GRIK3*** *GRID1* ***NEURL1*** *P2RX1* ***UNC13A*** <u>*ADCY8*</u> *DISC1* ***PRKCB*** *ADGRB1 PRKN NTRK1 CYFIP1 APP*
Movement of cell or subcellular component	0.0001	52	2139	*DNAH9* ***EFNB1*** *KCNE5* ***DYNC1H1*** *KIF13B* <u>*TRAF3IP1*</u> *CYFIP1* ***TBX5 GATA3*** *PPARG* ***CDH13 PTK2*** *ADGRB1 NEO1* <u>*HDAC4*</u> *CRMP1 NOX4* ***CACNA1I MYH14 NEURL1*** *BVES DPYSL3 APOA1 PREX1 BCAS3 APP* ***ITGA9*** *GNA12* ***DPYSL4*** *DISC1* ***NFASC ATP7A DRGX*** *DPCD CATSPER4 NTRK1* ***MAP2K3 PTPRC*** *ITGA11 SKAP1 ADAMTS12 RNF207* ***LDLRAD4*** *MYO18A* ***CDK6 FGF13 LYST RANBP9 KIFAP3*** *TNPO1 SLC7A5 INPP5D*
Multi-organism process	0.0402	48	2827	***ATG16L1 NLRC4*** *MST1R* ***SERPINB9*** *PRKN* ***IFNLR1*** *RXRA* <u>*HDAC4*</u> ***MLH1 BRINP1 PTPRC*** *TNPO1 RCOR1 USP42* ***EIF4H NEURL1*** *P2RX1* ***FAM20A*** *ACVR2A CITED1* ***HERC2*** *LOXL1 RPTOR APP TDRD9* ***CTBP1*** *CTSB DPCD* ***TOPAZ1*** *NAALADL2 DEFB123 ADGRB1 GRID1 CATSPER4 SND1* ***TXNRD3*** <u>*TRAF3IP1*</u> *ITSN1* ***CDK6 GATA3 MORC2 NPLOC4 LYST NUP188*** *HTATSF1* ***ATP7A RUVBL1*** *SIGIRR*
Muscle organ development	0.0082	14	404	*MYOM2 MYOM1 BVES* ***TBX5*** <u>*HDAC4*</u> ***NEURL1*** *UNC45A* ***ZFHX3*** *NR1D2 ADGRB1 RXRA* ***MYH14*** *ITGA11* <u>*COL6A3*</u>
Muscle structure development	0.0407	16	645	*MYOM2 MYOM1 BVES* ***TBX5*** <u>*HDAC4*</u> *NOX4* ***NEURL1*** *UNC45A* ***ZFHX3*** *TSC1 NR1D2 ADGRB1 RXRA* ***MYH14*** *ITGA11* <u>*COL6A3*</u>
Negative regulation of cell communication	0.0134	31	1440	***NEURL1*** *OTUD4 TSC1 PEAR1 AXIN1 APOA1* ***ITPR1*** *ADAMTS12* ***WWC2 LDLRAD4*** *PRKN INSIG1* ***PTPRC*** <u>*RAPGEF1*</u> ***GATA3*** <u>*ADCY8*</u> *INPP5D SIGIRR ADAMTSL2 ZNF536* <u>*TRAF3IP1*</u> ***RANBP9*** *EPN2* ***CGNL1*** *PPARG* ***NPLOC4 NDUFA13 MDFI GRIK3 PRKCB*** *CITED1*
Negative regulation of cell differentiation	0.0043	22	774	*PPARG* ***CDK6*** *ADAMTS7 ADAMTS12* <u>*HDAC4*</u> ***BRINP1*** *DPYSL3 CITED1* ***KLK8 CDK5RAP2*** *APP TSC1 INPP5D* ***PTK2*** *INSIG1 ZNF536 CRMP1* ***GATA3*** *FOXA2* ***LDLRAD4 FGF13 TBX5***
Negative regulation of cell proliferation	0.0017	23	737	*TNFRSF21* ***TBX5 GATA3 NEURL1*** *PPARG* ***CDH13*** *APP NTRK1* <u>*HDAC4*</u> ***KIFAP3*** *NOX4* <u>*RAPGEF1*</u> ***PEMT*** *TSC1 INPP5D* ***HNF4A CDK6 PBRM1 IFNLR1 TENM1*** *FRK* ***CTBP1*** *ADGRB1*

Negative regulation of cellular metabolic process	0.0475	46	2724	*KAT6A* ***SUDS3*** *PPARG* ***ZFHX3*** ***SERPINB9*** *NR1D2 PRKN ZNF536* <u>*HDAC4*</u> ***PTPRC*** *RCOR1* ***HNF4A*** *FRK CITED1* ***MORC2*** ***WWC2*** ***NDUFA13*** *RXRA* ***CLEC16A*** *LMCD1 ACADVL* ***MLH1*** ***CDK6*** <u>*RAPGEF1*</u> ***GATA3*** ***MAPK10*** *PPP2R5D SORBS3 KDM4B TRIM29 RPTOR APP* ***CTBP1*** <u>*COL6A3*</u> *TSC1 INPP5D* ***PTK2*** *ADGRB1 SIGIRR INSIG1* ***RANBP9*** *THRAP3* ***LDLRAD4*** <u>*TRAF3IP1*</u> ***MDFI*** *FOXA2*
Negative regulation of cytokine-mediated signaling pathway	0.0138	5	59	*OTUD4 APOA1* ***PTPRC*** *SIGIRR PPARG*
Negative regulation of developmental process	0.0018	28	1017	*TNFRSF21 PPARG* ***TBX5*** ***CDK6*** ***GATA3*** *ADAMTS7 ADAMTS12 ADGRB1* <u>*HDAC4*</u> ***BRINP1*** *DPYSL3 CITED1* ***KLK8*** ***CDK5RAP2*** *APP TSC1 INPP5D* ***PTK2*** *INSIG1 ZNF536* <u>*TRAF3IP1*</u> *CRMP1 EPN2 FOXA2* ***WWC2*** ***LDLRAD4*** ***FGF13*** *PRKN*
Negative regulation of epithelial to mesenchymal transition	0.0437	3	28	*FOXA2* ***LDLRAD4*** ***TBX5***
Negative regulation of glucokinase activity	0.0226	2	5	*PRKN FOXA2*
Negative regulation of hexokinase activity	0.0226	2	5	*PRKN FOXA2*
Negative regulation of monocyte differentiation	0.0354	2	7	***CDK6*** *INPP5D*
Negative regulation of multicellular organismal process	0.0041	31	1286	*TNFRSF21* ***TBX5*** ***CDK6*** ***GATA3*** *APOA1 PPARG ADAMTS7 ADAMTS12 ADGRB1* <u>*HDAC4*</u> ***BRINP1*** ***PTPRC*** *DPYSL3 IL18R1 CITED1* ***KLK8*** ***CDK5RAP2*** *APP TSC1 INPP5D* ***PTK2*** *SIGIRR ZNF536* <u>*TRAF3IP1*</u> *CRMP1 EPN2 FOXA2* ***WWC2*** ***LDLRAD4*** ***NPLOC4*** ***FGF13***
Negative regulation of myelination	0.0354	2	7	*TNFRSF21* ***KLK8***
Negative regulation of nervous system development	0.0085	12	314	*TNFRSF21* ***BRINP1*** *DPYSL3* ***KLK8*** ***CDK5RAP2*** *APP TSC1* ***PTK2*** *ZNF536* <u>*TRAF3IP1*</u> *CRMP1* ***FGF13***
Negative regulation of neurogenesis	0.0328	10	294	***BRINP1*** *DPYSL3* ***KLK8*** ***CDK5RAP2*** *APP TSC1* ***PTK2*** *ZNF536 CRMP1* ***FGF13***
Negative regulation of neuron differentiation	0.0222	9	225	*DPYSL3* ***KLK8*** ***CDK5RAP2*** *APP TSC1* ***PTK2*** *ZNF536 CRMP1* ***FGF13***
Negative regulation of response to cytokine stimulus	0.0162	5	63	*OTUD4 APOA1* ***PTPRC*** *SIGIRR PPARG*
Negative regulation of response to stimulus	0.0418	33	1764	***NEURL1*** *OTUD4 TSC1 PEAR1 AXIN1* ***CDK6*** *APOA1* ***ITPR1*** *ADAMTS12* ***WWC2*** ***LDLRAD4*** *PRKN INSIG1* ***PTPRC*** <u>*RAPGEF1*</u> ***GATA3*** ***KLK8*** *PPARG*

				INPP5D SIGIRR ADAMTSL2 ZNF536 TRAF3IP1 **RANBP9** EPN2 **CGNL1** **SERPINB9** **NPLOC4** **NDUFA13** **MDFI** FOXA2 **PRKCB** CITED1
Negative regulation of signal transduction	0.0138	29	1321	**NEURL1** OTUD4 TSC1 PEAR1 AXIN1 APOA1 **ITPR1** ADAMTS12 **WWC2** **LDLRAD4** PRKN INSIG1 **PTPRC** RAPGEF1 **GATA3** INPP5D SIGIRR ADAMTSL2 ZNF536 TRAF3IP1 **RANBP9** EPN2 **CGNL1** PPARG **NPLOC4** **NDUFA13** **MDFI** **PRKCB** CITED1
Negative regulation of signaling	0.0137	31	1444	**NEURL1** OTUD4 TSC1 PEAR1 AXIN1 APOA1 **ITPR1** ADAMTS12 **WWC2** **LDLRAD4** PRKN INSIG1 **PTPRC** RAPGEF1 **GATA3** ADCY8 INPP5D SIGIRR ADAMTSL2 ZNF536 TRAF3IP1 **RANBP9** EPN2 **CGNL1** PPARG **NPLOC4** **NDUFA13** **MDFI** **GRIK3** **PRKCB** CITED1
Nervous system development	0.0019	51	2474	APP **SUDS3** **TENM1** **GPM6B** **BRINP1** **EFNB1** **NEURL1** **KLK8** **ZFHX3** TNFRSF21 NTRK1 CYFIP1 **UNC13A** KIF13B NEO1 CRMP1 SLC7A5 RAPGEF1 **GATA3** DPYSL3 MTR APOA1 PREX1 CITED1 **FGF13** PPARG AGTPBP1 **CDK5RAP2** **DPYSL4** RDH13 DISC1 **NFASC** **TSHR** **DRGX** TSC1 DPCD **PTK2** **ARNT2** ADGRB1 PRKN INSC ZNF536 TRAF3IP1 ITSN1 FOXA2 **CDK6** **MDGA2** **ATP7A** **RANBP9** HDAC4 PPP2R5D
Neurogenesis	0.0003	42	1683	**TENM1** **GPM6B** **BRINP1** **EFNB1** **KLK8** **ZFHX3** NTRK1 CYFIP1 **UNC13A** KIF13B NEO1 CRMP1 RAPGEF1 **GATA3** **NEURL1** DPYSL3 MTR APOA1 PREX1 PPARG AGTPBP1 **CDK5RAP2** APP TNFRSF21 **DPYSL4** RDH13 DISC1 **NFASC** **TSHR** **DRGX** TSC1 **PTK2** PRKN ZNF536 ITSN1 FOXA2 **CDK6** **FGF13** **MDGA2** **ATP7A** **RANBP9** ADGRB1
Neuron development	0.0003	33	1154	**TENM1** **GPM6B** **EFNB1** **KLK8** NTRK1 CYFIP1 **UNC13A** KIF13B NEO1 CRMP1 RAPGEF1 **GATA3** **NEURL1** DPYSL3 MTR APOA1 PREX1 APP TNFRSF21 **DPYSL4** RDH13 DISC1 **NFASC** **TSHR** **DRGX** TSC1 **PTK2** PRKN ITSN1 **FGF13** **ATP7A** **RANBP9** ADGRB1
Neuron differentiation	0.0001	40	1412	**TENM1** **GPM6B** **BRINP1** **EFNB1** **KLK8** **ZFHX3** NTRK1 CYFIP1 **UNC13A** KIF13B NEO1 CRMP1 RAPGEF1 **GATA3** **NEURL1** DPYSL3 MTR APOA1 PREX1 AGTPBP1 **CDK5RAP2** APP TNFRSF21 **DPYSL4** RDH13 DISC1 **NFASC** **TSHR** **DRGX** TSC1 **PTK2** PRKN ZNF536 ITSN1 FOXA2 **FGF13** **MDGA2** **ATP7A** **RANBP9** ADGRB1

Neuron projection development	0.0002	31	1008	***GPM6B EFNB1 KLK8*** *NTRK1 CYFIP1* ***UNC13A*** *KIF13B NEO1 CRMP1 RAPGEF1* ***GATA3 NEURL1*** *DPYSL3 MTR APOA1 PREX1 APP TNFRSF21* ***DPYSL4*** *DISC1* ***NFASC TSHR DRGX*** *TSC1* ***PTK2*** *PRKN ITSN1* ***FGF13 ATP7A RANBP9*** *ADGRB1*
Neuron projection guidance	0.0043	12	275	***EFNB1*** *CYFIP1 NEO1 CRMP1* ***GATA3*** *APP* ***DPYSL4 NFASC DRGX*** *NTRK1* ***RANBP9 PTK2***
Neuron projection morphogenesis	0.0043	20	666	***EFNB1 KLK8*** *CYFIP1* ***UNC13A*** *KIF13B NEO1 CRMP1* ***GATA3*** *APP* ***DPYSL4*** *DISC1* ***NFASC DRGX PTK2*** *PRKN NTRK1* ***FGF13 ATP7A RANBP9*** *ADGRB1*
Nitrogen compound transport	0.0375	45	2592	***GPM6B SEC31B*** *TNPO1 SCAMP1* ***NLRC4 NUP188*** *TNFRSF21* ***GATA3*** *CRTAM APOA1 PPARG* ***STXBP4*** *PRKN* ***NDUFA13*** *INSIG1 KLHL20* ***ATG16L1 HNF4A*** *SLC7A5 AAGAB P2RX1* ***MAPK10*** *SLC16A10* ***SIL1*** *BCAS3* ***LYST UEVLD*** *ADCY8 AGAP1 ITSN1 DENND1B MYOM1* ***LMAN2L MCU*** *TSC1* ***NPLOC4*** *MYO18A* ***TENM1*** *FOXA2* ***HERC2*** *ACAA1 ATP6V0D2* ***ITPR1 PRKCB*** *KIF13B*
Norepinephrine metabolic process	0.0087	3	12	***GATA3*** *PRKN* ***ATP7A***
Organelle organization	0.0150	68	4098	*MYOM2* ***SEC31B*** *KAT6A* ***ATG16L1*** *MYOM1 ATG2A* ***SUDS3 LMAN2L*** *KDM4B MAU2* ***UNC13A*** *ERAL1* ***ATG12*** *NDUFAF6* ***PBRM1*** *DEUP1* ***RUVBL1*** *PRKN* ***DYNC1H1*** *TRAF3IP1 HDAC4 RCOR1* ***MYH14*** *APOA1 SORBS3* ***MORC2 CDK5RAP2*** *TSC1* ***PRKCB NDUFA13*** *SUPT3H MBTD1* ***CLEC16A*** *CRMP1* ***MLH1*** *NOX4* ***GATA3 NEURL1*** *DPYSL3 PREX1 FOXA2 AGTPBP1 BCAS3* ***LYST*** *TDRD9 DISC1 ABLIM2* ***PTK2 GPM6B RNF4 CGNL1*** *SKAP1* ***CTBP1*** *TRAPPC12 INSIG1 MYO18A CYFIP1* ***TENM1 FGF13 ATP7A NPLOC4 RANBP9*** *ACAA1* ***KIFAP3*** *KLHL20 TNPO1 PPP1R13B ATP6V0D2*
Organic substance transport	0.0250	52	3011	***GPM6B SEC31B*** *TNPO1 SCAMP1* ***NLRC4 NUP188 SLC25A14*** *APOA1 TNFRSF21 PPARG* ***GATA3*** *CRTAM* ***STXBP4*** *PRKN* ***NDUFA13*** *INSIG1 KLHL20* ***ATG16L1 HNF4A*** *SLC7A5 AAGAB P2RX1* ***MAPK10*** *SLC16A10* ***SIL1*** *SLC26A10 BCAS3* ***LYST UEVLD*** *SLC5A10 ADCY8 AGAP1 TSC1* ***PRKCB MFSD4A*** *ITSN1 DENND1B MYOM1* ***LMAN2L MCU NPLOC4*** *MYO18A* ***TENM1*** *FOXA2 SLC22A18* ***HERC2*** *ACAA1 ATP6V0D2* ***ITPR1*** *RXRA KIF13B ACACA*

Peptide transport	0.0173	42	2228	**SEC31B** TNPO1 SCAMP1 **NLRC4** **NUP188** TNFRSF21 **GATA3** CRTAM APOA1 PPARG **STXBP4** PRKN **NDUFA13** INSIG1 **GPM6B** KLHL20 **ATG16L1** **HNF4A** AAGAB **MAPK10** **SIL1** BCAS3 **LYST** **UEVLD** ADCY8 AGAP1 ITSN1 DENND1B MYOM1 **LMAN2L** **MCU** TSC1 **NPLOC4** MYO18A **TENM1** FOXA2 **HERC2** ACAA1 ATP6V0D2 **ITPR1** **PRKCB** KIF13B
Peptidyl-amino acid modification	0.0049	31	1312	KAT6A FRK LOXL1 PLOD2 **STK32C** **PRKCB** **CSNK1G1** **PTK2** **RUVBL1** HDAC4 **PTPRC** APOA1 SUPT3H NTRK1 **MAP2K3** NOX4 **GATA3** **NEURL1** PPP2R5D KDM4B **DSTYK** AGTPBP1 APP MST1R RPTOR **CTBP1** **TENM1** **ATP7A** AGBL1 AXIN1 **NUP188**
Peptidyl-threonine modification	0.0411	6	127	**CSNK1G1** **PRKCB** PPP2R5D RPTOR APP AXIN1
Peptidyl-threonine phosphorylation	0.0349	6	119	**CSNK1G1** **PRKCB** PPP2R5D RPTOR APP AXIN1
Phosphorylation	0.0455	44	2570	**MAP2K3** **CDK6** **MAPK10** FRK PPP2R5D RPTOR **STK32C** **PRKCB** **CSNK1G1** **PTK2** PRKN NTRK1 **LAT** **PTPRC** AXIN1 **FAM20A** **FGF13** APP MST1R **GRK5** HDAC4 NOX4 RAPGEF1 **NEURL1** **PRKD3** APOA1 SORBS3 ACVR2A **DSTYK** **NME7** TSC1 TPK1 ITSN1 **RANBP9** GAREM1 **LDLRAD4** TRAF3IP1 **TENM1** FOXA2 ADCY8 **ATP7A** **NUP188** **CTBP1** **NDUFA13**
Plasma membrane bounded cell projection assembly	0.0400	15	584	ABLIM2 CYFIP1 **CDH13** HDAC4 **NEURL1** DPYSL3 BCAS3 DISC1 **ATP7A** TRAF3IP1 **TENM1** **KIFAP3** TNPO1 **CDK5RAP2** **DYNC1H1**
Plasma membrane bounded cell projection morphogenesis	0.0051	20	680	**EFNB1** **KLK8** CYFIP1 **UNC13A** KIF13B NEO1 CRMP1 **GATA3** APP **DPYSL4** DISC1 **NFASC** **DRGX** **PTK2** PRKN NTRK1 **FGF13** **ATP7A** **RANBP9** ADGRB1
Plasma membrane bounded cell projection organization	0.0002	41	1552	**GPM6B** **EFNB1** **KLK8** ABLIM2 NTRK1 TRAF3IP1 CYFIP1 **UNC13A** **CDH13** KIF13B NEO1 HDAC4 CRMP1 RAPGEF1 **GATA3** **NEURL1** DPYSL3 MTR APOA1 PREX1 BCAS3 APP TNFRSF21 **DPYSL4** DISC1 **NFASC** **ATP7A** **TSHR** **DRGX** TSC1 **PTK2** PRKN ITSN1 **TENM1** **FGF13** **RANBP9** **KIFAP3** TNPO1 **CDK5RAP2** ADGRB1 **DYNC1H1**
Positive regulation of catalytic activity	0.0138	32	1514	**MAP2K3** **NLRC4** RPTOR **LAT** **NDUFA13** AXIN1 RAPGEF1 APOA1 PPARG BCAS3 MST1R **ATP7A** TSC1 NTRK1 **MYO9A** **PTPRC** NOX4 **NEURL1** P2RX1 **FAM20A** **MAPK10** PREX1 **FGF13** APP AGAP1 **ADAP2** ITSN1 DENND1B **DSTYK** **PTK2** **TENM1** ADCY8

Positive regulation of cell adhesion	0.0192	14	465	COL26A1 FOXA2 **CDK6** APOA1 SKAP1 TSC1 **KIFAP3** **PTPRC** **EFNB1** **GATA3** PREX1 DISC1 **CDH13** **ZFHX3**
Positive regulation of cell communication	0.0043	41	1937	**MAP2K3** **RRAGB** MST1R **CSNK1G1** NTRK1 APOA1 ACVR2A **CDH13** RPTOR **RUVBL1** PRKN NEO1 LMCD1 **PTPRC** NOX4 RAPGEF1 **NEURL1** **MAPK10** IL18R1 SORBS3 **UNC13A** SKAP1 ADCY8 DISC1 **PRKCB** ITSN1 **LAT** CYFIP1 APP **CLEC16A** EPN2 **GATA3** **DSTYK** GAREM1 **MCU** **PTK2** **TENM1** AXIN1 RNF207 PPP1R13B CITED1
Positive regulation of cell projection organization	0.0150	13	395	NTRK1 **UNC13A** HDAC4 RAPGEF1 **NEURL1** DPYSL3 BCAS3 DISC1 **ATP7A** PRKN ITSN1 CYFIP1 **TENM1**
Positive regulation of cell-matrix adhesion	0.0090	5	52	**CDK6** TSC1 DISC1 SKAP1 **CDH13**
Positive regulation of cell-substrate adhesion	0.0041	8	120	COL26A1 **CDK6** APOA1 TSC1 PREX1 DISC1 SKAP1 **CDH13**
Positive regulation of cellular component biogenesis	0.0226	16	586	APOA1 SORBS3 SKAP1 TSC1 HDAC4 NOX4 **NEURL1** DPYSL3 BCAS3 **ATP7A** ADGRB1 NTRK1 CYFIP1 **CDK5RAP2** **DYNC1H1** **TENM1**
Positive regulation of cellular component organization	0.0064	31	1344	NTRK1 APOA1 SORBS3 **UNC13A** **MORC2** SKAP1 TSC1 PRKN **CLEC16A** HDAC4 **PTPRC** NOX4 RAPGEF1 **GATA3** **NEURL1** DPYSL3 PREX1 PPARG BCAS3 DISC1 **ATP7A** ADGRB1 ITSN1 CYFIP1 **CDK5RAP2** APP **CTBP1** MYO18A **DYNC1H1** **TENM1** PPP1R13B
Positive regulation of cellular metabolic process	0.0035	64	3482	**MAP2K3** **RNF4** KAT6A **NLRC4** APOA1 PPARG **ZFHX3** RPTOR NR1D2 PRKN NTRK1 **LAT** **NDUFA13** THRAP3 HDAC4 **TBX5** **HNF4A** AXIN1 **GATA3** **FAM20A** FOXA2 CITED1 **MORC2** **CDK5RAP2** SKAP1 BCAS3 APP MST1R OTUD4 **ARNT2** ETV4 RXRA INSIG1 **CLEC16A** **MLH1** **PTPRC** **ATG16L1** NOX4 RAPGEF1 **NEURL1** P2RX1 **MAPK10** BVES PPP2R5D SORBS3 **FGF13** **PEMT** DISC1 TSC1 **PRKCB** **PTK2** **RUVBL1** **BRF1** ITSN1 ACVR2A **DSTYK** **CDH13** GAREM1 **CTBP1** ADGRB1 **TENM1** ADCY8 SUPT3H ACACA
Positive regulation of cellular protein metabolic process	0.0400	32	1680	**MAP2K3** **NLRC4** RPTOR PRKN NTRK1 **LAT** **NDUFA13** HDAC4 AXIN1 **FAM20A** PPARG APP MST1R **PTPRC** NOX4 RAPGEF1 **GATA3** **NEURL1** P2RX1 **MAPK10** PPP2R5D SORBS3 **FGF13** DISC1 **PTK2** RXRA ACVR2A **DSTYK** GAREM1 **CTBP1** **TENM1** ADCY8
Positive regulation of cytoskeleton organization	0.0348	9	250	APOA1 SORBS3 NOX4 BCAS3 TSC1 **CDK5RAP2** **DYNC1H1** CYFIP1 **TENM1**

Positive regulation of Fc receptor mediated stimulatory signaling pathway	0.0411	2	8	***PTPRC*** *RAPGEF1*
Positive regulation of Fc-gamma receptor signaling pathway involved in phagocytosis	0.0300	2	6	***PTPRC*** *RAPGEF1*
Positive regulation of filopodium assembly	0.0101	4	30	***NEURL1*** *DPYSL3 BCAS3* ***TENM1***
Positive regulation of intracellular signal transduction	0.0233	25	1133	***MAP2K3 RRAGB*** *MST1R NTRK1 APOA1* ***CDH13*** *RPTOR PRKN LMCD1* ***PTPRC*** *NOX4 RAPGEF1* ***MAPK10*** *IL18R1 SORBS3* ***PRKCB*** *ITSN1 APP* ***CLEC16A GATA3 DSTYK*** *GAREM1* ***PTK2 TENM1*** *AXIN1*
Positive regulation of kinase activity	0.0077	18	604	***MAP2K3*** *RPTOR* ***LAT*** *AXIN1 MST1R* ***PTPRC*** *NOX4* ***NEURL1 FAM20A MAPK10 FGF13*** *ITSN1* ***DSTYK PTK2 TENM1*** *ADCY8 RAPGEF1 NTRK1*
Positive regulation of macromolecule metabolic process	0.0104	61	3498	***MAP2K3 RNF4*** *KAT6A* ***NLRC4*** *PPARG* ***ZFHX3*** *RPTOR NR1D2 PRKN NTRK1* ***LAT*** *AXIN1* ***NDUFA13*** *THRAP3 HDAC4* ***TBX5 HNF4A GATA3 FAM20A*** *FOXA2 CITED1* ***MORC2 CDK5RAP2*** *SKAP1 BCAS3 APP MST1R OTUD4* ***ARNT2*** *ETV4 RXRA INSIG1* ***MLH1 PTPRC*** *NOX4* ***CDK6*** *RAPGEF1* ***NEURL1*** *P2RX1* ***MAPK10*** *BVES PPP2R5D SORBS3* ***FGF13 PEMT*** *DISC1* ***PRKCB PTK2 RUVBL1 BRF1*** *MYOM1 ACVR2A* ***DSTYK CDH13*** *GAREM1 RNF207* ***CTBP1 SERPINB9 TENM1*** *ADCY8 SUPT3H*
Positive regulation of metabolic process	0.0014	71	3789	***MAP2K3 RNF4*** *KAT6A* ***NLRC4*** *APOA1 PPARG* ***ZFHX3*** *RPTOR NR1D2 PRKN NTRK1* ***LAT*** *AXIN1* ***NDUFA13*** *THRAP3 HDAC4* ***TBX5 HNF4A GATA3 FAM20A*** *FOXA2 CITED1* ***MORC2 CDK5RAP2*** *SKAP1 BCAS3 APP MST1R OTUD4* ***ATP7A ARNT2*** *ETV4 RXRA INSIG1* ***CLEC16A MLH1 PTPRC ATG16L1*** *NOX4* ***CDK6*** *RAPGEF1* ***NEURL1*** *P2RX1* ***MAPK10*** *BVES PPP2R5D SORBS3* ***FGF13 PEMT*** *DISC1* ***TSHR*** *TSC1* ***PRKCB PTK2 RUVBL1 BRF1 DYNC1H1*** *ITSN1 MYOM1 ACVR2A* ***DSTYK CDH13*** *GAREM1 RNF207* ***CTBP1 SERPINB9*** *ADGRB1* ***TENM1*** *ADCY8 SUPT3H ACACA*
Positive regulation of molecular function	0.0083	39	1901	***MAP2K3 NLRC4*** *RPTOR* ***LAT NDUFA13*** *AXIN1 RAPGEF1 IL18R1 APOA1 MAU2 PPARG BCAS3 MST1R* ***ATP7A*** *TSC1 PRKN NTRK1* ***MYO9A PTPRC*** *NOX4* ***GATA3 NEURL1*** *P2RX1* ***FAM20A MAPK10*** *PREX1* ***FGF13*** *APP ADCY8 AGAP1* ***PRKCB ADAP2*** *ITSN1 DENND1B RNF207 HDAC4* ***DSTYK PTK2 TENM1***

Positive regulation of nitrogen compound metabolic process	0.0303	56	3351	***MAP2K3*** ***RNF4*** *KAT6A* ***NLRC4*** *PPARG* ***ZFHX3*** *RPTOR* *NR1D2* *PRKN* *NTRK1* ***LAT*** *AXIN1* ***NDUFA13*** *THRAP3* <u>*HDAC4*</u> ***TBX5*** ***HNF4A*** ***GATA3*** ***FAM20A*** *FOXA2* *CITED1* ***MORC2*** ***CDK5RAP2*** *SKAP1* *BCAS3* *APP* *MST1R* *OTUD4* ***ARNT2*** *ETV4* *RXRA* *INSIG1* ***MLH1*** ***PTPRC*** *NOX4* <u>*RAPGEF1*</u> ***NEURL1*** *P2RX1* ***MAPK10*** *PPP2R5D* *SORBS3* ***FGF13*** ***PEMT*** *DISC1* ***PRKCB*** ***PTK2*** ***RUVBL1*** ***BRF1*** *ACVR2A* ***DSTYK*** ***CDH13*** <u>*GAREM1*</u> ***CTBP1*** ***TENM1*** <u>*ADCY8*</u> *SUPT3H*
Positive regulation of peptidyl-threonine phosphorylation	0.0499	3	30	*RPTOR* *APP* *AXIN1*
Positive regulation of phagocytosis	0.0370	5	84	*APOA1* ***PTPRC*** <u>*RAPGEF1*</u> *PPARG* *MYO18A*
Positive regulation of plasma membrane bounded cell projection assembly	0.0074	7	105	<u>*HDAC4*</u> ***NEURL1*** *DPYSL3* *BCAS3* ***ATP7A*** *CYFIP1* ***TENM1***
Positive regulation of protein kinase activity	0.0159	16	557	***MAP2K3*** *RPTOR* ***LAT*** *AXIN1* *MST1R* ***PTPRC*** *NOX4* ***NEURL1*** ***FAM20A*** ***MAPK10*** ***FGF13*** ***PTK2*** ***TENM1*** <u>*ADCY8*</u> <u>*RAPGEF1*</u> *NTRK1*
Positive regulation of protein metabolic process	0.0496	33	1796	***MAP2K3*** ***NLRC4*** *RPTOR* *PRKN* *NTRK1* ***LAT*** *AXIN1* ***NDUFA13*** <u>*HDAC4*</u> ***FAM20A*** *PPARG* *APP* *MST1R* ***PTPRC*** *NOX4* <u>*RAPGEF1*</u> ***GATA3*** ***NEURL1*** *P2RX1* ***MAPK10*** *PPP2R5D* *SORBS3* ***FGF13*** ***PEMT*** *DISC1* ***PTK2*** *RXRA* *ACVR2A* ***DSTYK*** <u>*GAREM1*</u> ***CTBP1*** ***TENM1*** <u>*ADCY8*</u>
Positive regulation of protein modification process	0.0410	26	1279	***MAP2K3*** *RPTOR* *NTRK1* ***LAT*** <u>*HDAC4*</u> *AXIN1* ***FAM20A*** *APP* *MST1R* ***PTPRC*** *NOX4* <u>*RAPGEF1*</u> ***GATA3*** ***NEURL1*** ***MAPK10*** *PPP2R5D* *SORBS3* ***FGF13*** *PRKN* *ACVR2A* ***DSTYK*** <u>*GAREM1*</u> ***CTBP1*** ***PTK2*** ***TENM1*** <u>*ADCY8*</u>
Positive regulation of response to stimulus	0.0069	50	2621	***MAP2K3*** ***RRAGB*** ***NLRC4*** *MST1R* *OTUD4* ***CSNK1G1*** *NTRK1* *DENND1B* ***PTPRC*** *CRTAM* *IL18R1* *APOA1* *ACVR2A* ***CDH13*** *SKAP1* *RPTOR* *SUSD4* ***RUVBL1*** *PRKN* *NEO1* *LMCD1* ***MLH1*** *NOX4* <u>*RAPGEF1*</u> ***GATA3*** ***NEURL1*** ***MAPK10*** *SORBS3* ***UNC13A*** *TNFRSF21* <u>*ADCY8*</u> *DISC1* ***ATP7A*** ***PRKCB*** *ITSN1* ***LAT*** *CYFIP1* *APP* ***CLEC16A*** *EPN2* ***DSTYK*** <u>*GAREM1*</u> ***PTK2*** ***NPLOC4*** ***TENM1*** *AXIN1* *PPP1R13B* *CITED1* *CTSB* *INPP5D*
Positive regulation of retrograde transport. endosome to Golgi	0.0054	2	2	*EIPR1* *PRKN*
Positive regulation of signal transduction	0.0051	38	1762	***MAP2K3*** ***RRAGB*** *MST1R* ***CSNK1G1*** *NTRK1* *APOA1* *ACVR2A* ***CDH13*** *RPTOR* ***RUVBL1*** *PRKN* *NEO1* *LMCD1* ***PTPRC*** *NOX4* <u>*RAPGEF1*</u> ***NEURL1*** ***MAPK10***

				IL18R1 SORBS3 ***UNC13A*** *SKAP1 DISC1* ***PRKCB*** *ITSN1* ***LAT*** *CYFIP1 APP* ***CLEC16A*** *EPN2* ***GATA3 DSTYK*** <u>*GAREM1*</u> ***PTK2 TENM1*** *AXIN1 PPP1R13B CITED1*
Positive regulation of signaling	0.0033	42	1945	***MAP2K3 RRAGB*** *MST1R* ***CSNK1G1*** *NTRK1 APOA1 ACVR2A* ***CDH13*** *RPTOR* ***RUVBL1*** *PRKN NEO1 LMCD1* ***PTPRC*** *NOX4* <u>*RAPGEF1*</u> ***NEURL1 MAPK10*** *BVES IL18R1 SORBS3* ***UNC13A*** *SKAP1* <u>*ADCY8*</u> *DISC1* ***PRKCB*** *ITSN1* ***LAT*** *CYFIP1 APP* ***CLEC16A*** *EPN2* ***GATA3 DSTYK*** <u>*GAREM1*</u> ***MCU PTK2 TENM1*** *AXIN1 RNF207 PPP1R13B CITED1*
Positive regulation of stress fiber assembly	0.0437	4	56	*APOA1 SORBS3 NOX4 TSC1*
Positive regulation of supramolecular fiber organization	0.0499	8	227	*APOA1 SORBS3 NOX4 TSC1* ***CDK5RAP2*** *APP CYFIP1* ***TENM1***
Positive regulation of transferase activity	0.0180	18	682	***MAP2K3*** *RPTOR* ***LAT*** *AXIN1 MST1R* ***PTPRC*** *NOX4* ***NEURL1 FAM20A MAPK10 FGF13*** *ITSN1* ***DSTYK PTK2 TENM1*** <u>*ADCY8*</u> <u>*RAPGEF1*</u> *NTRK1*
Protein deglutamylation	0.0411	2	8	*AGTPBP1 AGBL1*
Protein K6-linked ubiquitination	0.0411	2	8	*PRKN* ***RNF4***
Protein localization	0.0153	53	2988	***SEC31B*** *TNPO1 SCAMP1* ***NLRC4 NUP188*** *TNFRSF21 PRKN* ***GATA3*** *CRTAM APOA1 MAU2 PPARG* ***STXBP4 NDUFA13*** *INSIG1* ***GPM6B KIFAP3*** *KLHL20* ***ATG16L1 HNF4A*** *AAGAB* ***MAPK10 SIL1*** *TRIM29 SKAP1 BCAS3* ***LYST UEVLD*** <u>*ADCY8*</u> *AGAP1 DISC1* ***NFASC*** *ITSN1 DENND1B MYOM1* ***RRAGB LMAN2L MCU*** *TSC1 TRAPPC12* ***NPLOC4*** *MYO18A* ***TENM1*** *FOXA2* ***FGF13 HERC2*** *ACAA1 PPP1R13B* ***MDFI*** *ATP6V0D2* ***ITPR1 PRKCB*** *KIF13B*
Protein modification process	0.0411	69	4434	*EIPR1* ***MAP2K3*** *KLHL20 KAT6A* ***CDK6*** *USP42* ***MAPK10 SUDS3*** *FRK PPP2R5D LOXL1 RPTOR* ***ATG12*** *PLOD2 OTUD4* ***STK32C PRKCB CSNK1G1 PTK2 RUVBL1*** *ALG12 PRKN NTRK1* ***LAT*** <u>*HDAC4*</u> ***PTPRC*** *RCOR1 AXIN1* ***FAM20A*** *APOA1 KDM4B* ***HERC2 FGF13*** *APP MST1R SUPT3H* ***GRK5 RNF4 ATG16L1*** *NOX4* <u>*RAPGEF1*</u> ***GATA3 NEURL1*** *PTPN5* ***PRKD3*** *SORBS3 RNF170 ACVR2A* ***LRRC29*** *UBAC1* ***DSTYK*** *AGTPBP1* ***UEVLD*** *TSC1 ADGRB1 ZNRF1* ***FUT4 RANBP9*** <u>*GAREM1*</u> ***CTBP1 LDLRAD4*** <u>*TRAF3IP1*</u> ***TENM1*** <u>*ADCY8*</u> ***ATP7A*** *AGBL1* ***NUP188 FBXL16*** <u>*WDR20*</u>
Protein side chain deglutamylation	0.0300	2	6	*AGTPBP1 AGBL1*

Protein transport	0.0138	42	2187	**SEC31B** TNPO1 SCAMP1 **NLRC4** **NUP188** TNFRSF21 **GATA3** CRTAM APOA1 PPARG **STXBP4** PRKN **NDUFA13** INSIG1 **GPM6B** KLHL20 **ATG16L1** **HNF4A** AAGAB **MAPK10** **SIL1** BCAS3 **LYST** **UEVLD** ADCY8 AGAP1 ITSN1 DENND1B MYOM1 **LMAN2L** **MCU** TSC1 **NPLOC4** MYO18A **TENM1** FOXA2 **HERC2** ACAA1 ATP6V0D2 **ITPR1** **PRKCB** KIF13B
Protein-containing complex assembly	0.0437	38	2126	MYOM2 **SEC31B** **NLRC4** MYOM1 **EIF4H** APOA1 **UNC13A** ERAL1 NDUFAF6 **RUVBL1** **BRF1** AXIN1 SKAP1 **MCU** **LDLRAD4** TRAPPC12 RXRA KAT6A P2RX1 DPYSL3 PREX1 TSC1 **PTK2** **DYNC1H1** **RNF4** **CDK5RAP2** PRKN **NDUFA13** INSIG1 TRAF3IP1 CYFIP1 **TENM1** **FGF13** ADCY8 ACACA **RANBP9** **ATG16L1** **KIFAP3**
Protein-containing complex subunit organization	0.0300	44	2465	MYOM2 **SEC31B** **NLRC4** MYOM1 **EIF4H** APOA1 **UNC13A** ERAL1 PLOD2 NDUFAF6 **PBRM1** **RUVBL1** **BRF1** AXIN1 SKAP1 **MCU** **LDLRAD4** TRAPPC12 RXRA **CLEC16A** KAT6A P2RX1 DPYSL3 PREX1 TSC1 **PTK2** **DYNC1H1** **RNF4** **CDK5RAP2** PRKN **NDUFA13** INSIG1 TRAF3IP1 CYFIP1 **TENM1** **FGF13** ADCY8 **ATP7A** ACACA **RANBP9** **ATG16L1** **KIFAP3** MRPL19 MRPS6
Proteolysis	0.0222	38	1988	KLHL20 **NLRC4** USP42 **LRRC29** **FBXL16** **HERC2** OTUD4 CTSB **SERPINB9** **NPLOC4** PRKN **NDUFA13** PPARG SUSD4 ADAMTS12 SUPT3H **CLEC16A** P2RX1 **KLK8** AGTPBP1 ADAMTS7 **ADAMTS17** APP GNA12 DISC1 COL6A3 **PTK2** TRAPPC12 TMPRSS9 ZNRF1 ADAMTSL2 AGBL1 **RNF4** AXIN1 **GATA3** **SUDS3** WDR20 **RUVBL1**
Pyramidal neuron development	0.0482	2	9	DISC1 **ATP7A**
Regulation of actin filament-based process	0.0339	12	400	APOA1 SORBS3 TSC1 PRKN NOX4 PREX1 BCAS3 **GPM6B** RNF207 CYFIP1 **TENM1** **FGF13**
Regulation of anatomical structure morphogenesis	0.0223	25	1125	APOA1 **UNC13A** PPARG ADAMTS12 ADGRB1 KIF13B **TBX5** **HNF4A** **MYH14** **GATA3** BVES PREX1 CITED1 GNA12 DISC1 **PRKCB** **PTK2** PRKN RXRA CYFIP1 EPN2 DNMBP FOXA2 **FGF13** RNF207
Regulation of biological quality	0.0077	73	4319	APP **GPM6B** ACAA1 **CACNA1I** BVES APOA1 **UNC13A** PPARG RPTOR SLC12A9 ATP6V0D2 **ITPR1** **MCU** SYN2 SLC9C2 **ATP7A** KCNE5 PNPLA1 **DHRS7C** **TXNRD3** **HNF4A** ACVR2A FOXA2 **GRIK3** TSC1 **STXBP4** PRKN INSIG1 INSC NEO1 ACADVL **PTPRC** NOX4 **MYH14** **GATA3** **NEURL1** P2RX1

				MAPK10 *SLC16A10 IL18R1 PREX1* ***KLK8 PEMT*** *AGTPBP1 GNA12 ADCY8 RDH13 DISC1* ***TSHR PRKCB*** *INPP5D* ***PTK2*** *NR1D2 ADGRB1 GRID1 PEAR1 DPYD* ***DYNC1H1*** *NTRK1 ITSN1* ***LAT*** *CYFIP1 THRAP3* ***CDK6*** *DNMBP* ***FAM20A*** *RNF207* ***TENM1 FGF13*** *TNPO1 PPP1R13B RCOR1* ***MDFI***
Regulation of biosynthetic process	0.0345	73	4687	***RNF4*** *KAT6A* ***NLRC4 SUDS3*** *APOA1 FOXA2 CITED1 PPARG* ***ZFHX3*** *ZNF646 NR1D2* ***RUVBL1*** *ETV4 SUPT3H ZNF536 CYFIP1* ***MAP2K3*** *THRAP3 HDAC4* ***TBX5*** *RCOR1* ***HNF4A GATA3*** *FRK IL18R1 ACVR2A* ***MORC2 CDK5RAP2*** *SKAP1 BCAS3* ***WWC2*** *TSC1* ***ARNT2*** *PRKN* ***NDUFA13*** *RXRA INSIG1 NTRK1 MBTD1 NEO1 LMCD1 ACADVL* ***PTPRC*** *NOX4* ***CDK6 NEURL1 MAPK10*** *ZNF684 SORBS3* ***PHF20L1 PEMT*** *TRIM29 APP ADCY8* ***CTBP1 DRGX PRKCB*** *INPP5D PRDM10* ***BRF1*** *SIGIRR SND1 ZNF407 AXIN1* ***CDH13*** *RPTOR ADGRB1* ***TENM1 MDFI NUP188*** *HTATSF1* ***EIF4H*** *ACACA*
Regulation of cell adhesion	0.0406	18	765	*TNFRSF21 COL26A1* ***PTK2*** *FOXA2* ***CDK6*** *APOA1* ***CDH13*** *SKAP1 TSC1* ***KIFAP3 PTPRC EFNB1 GATA3*** *PREX1 BCAS3 DISC1* ***GPM6B ZFHX3***
Regulation of cell communication	0.0008	74	3903	***MAP2K3 RRAGB NEURL1 GRIK3*** *MST1R OTUD4 TSC1* ***CSNK1G1*** *GRID1 PEAR1 NTRK1 AXIN1 APP APOA1 ACVR2A* ***CDH13*** *RPTOR* ***ITPR1*** *ADAMTS12* ***WWC2 STXBP4 LDLRAD4 RUVBL1*** *PRKN INSIG1 NEO1 LMCD1* ***PTPRC*** *NOX4* ***HNF4A*** *RAPGEF1* ***GATA3*** *DNMBP P2RX1* ***MAPK10*** *IL18R1 SORBS3 PREX1* ***FGF13 UNC13A*** *SKAP1 GNA12 ADCY8 DISC1* ***PRKCB*** *INPP5D* ***PTK2*** *ADGRB1 SIGIRR ADAMTSL2 ZNF536 TRAF3IP1 ITSN1* ***LAT*** *CYFIP1* ***RANBP9 CLEC16A*** *EPN2* ***CGNL1*** *PPARG* ***DSTYK*** *GAREM1* ***MCU NPLOC4 NDUFA13 TENM1 MDFI*** *FOXA2 RNF207* ***MYO9A*** *KAT6A PPP1R13B CITED1* ***GRK5***
Regulation of cell development	0.0034	26	971	***BRINP1 ZFHX3*** *NTRK1 APOA1* ***UNC13A*** *KIF13B HDAC4 RAPGEF1* ***GATA3 NEURL1*** *DPYSL3 PREX1* ***KLK8*** *PPARG* ***CDK5RAP2*** *APP TNFRSF21 DISC1 TSC1* ***PTK2*** *PRKN ZNF536 ITSN1 CYFIP1 CRMP1* ***FGF13***
Regulation of cell differentiation	0.0077	40	1954	***BRINP1 GATA3 ZFHX3*** *NTRK1 PPARG* ***TBX5 CDK6*** *APOA1 ACVR2A* ***UNC13A*** *ADAMTS7 ADAMTS12 KIF13B HDAC4* ***PTPRC*** *RAPGEF1* ***NEURL1*** *DPYSL3 PREX1 CITED1* ***KLK8 CDK5RAP2*** *APP TNFRSF21 DISC1 TSC1 INPP5D* ***PTK2*** *NR1D2 ADGRB1 PRKN INSIG1 ZNF536 ITSN1 CYFIP1 CRMP1 FOXA2* ***LDLRAD4 FGF13 PRKCB***

Regulation of cell projection organization	0.0034	21	691	*NTRK1* ***UNC13A*** *KIF13B* <u>*HDAC4*</u> <u>*RAPGEF1*</u> ***GATA3*** ***NEURL1*** *DPYSL3* *PREX1* ***KLK8*** *BCAS3* *DISC1* ***ATP7A*** *TSC1* ***PTK2*** *PRKN* *ITSN1* *CYFIP1* *CRMP1* ***TENM1*** ***FGF13***
Regulation of cell proliferation	0.0119	36	1756	*TNFRSF21* ***PTK2*** ***TBX5*** ***GATA3*** ***NEURL1*** *PPARG* ***CDH13*** <u>*GAREM1*</u> *APP* *NTRK1* <u>*HDAC4*</u> ***KIFAP3*** ***PTPRC*** *NOX4* ***EFNB1*** ***CDK6*** <u>*RAPGEF1*</u> ***PEMT*** *RPTOR* *DISC1* ***ATP7A*** *TSC1* *INPP5D* ***ARNT2*** ***HNF4A*** ***PBRM1*** ***STXBP4*** ***GRK5*** ***IFNLR1*** ***TENM1*** *FRK* *CITED1* ***CTBP1*** *MST1R* ***TSHR*** *ADGRB1*
Regulation of cell-matrix adhesion	0.0043	8	122	***CDK6*** *TSC1* *BCAS3* *DISC1* *SKAP1* ***PTK2*** ***GPM6B*** ***CDH13***
Regulation of cell-substrate adhesion	0.0031	11	216	<u>*COL26A1*</u> ***CDK6*** *APOA1* *TSC1* *PREX1* *BCAS3* *DISC1* *SKAP1* ***PTK2*** ***GPM6B*** ***CDH13***
Regulation of cellular component biogenesis	0.0029	27	1002	*APP* *APOA1* *SORBS3* *SKAP1* *TSC1* ***LDLRAD4*** *PRKN* <u>*HDAC4*</u> *NOX4* ***NEURL1*** *DPYSL3* *PREX1* ***CDK5RAP2*** *BCAS3* ***ATP7A*** ***PTK2*** *ADGRB1* *NTRK1* *CYFIP1* ***GPM6B*** ***RNF4*** <u>*RAPGEF1*</u> *TRAPPC12* *INSIG1* ***DYNC1H1*** <u>*TRAF3IP1*</u> ***TENM1***
Regulation of cellular component organization	0.0054	51	2653	*APP* *RPTOR* *PRKN* *NTRK1* ***PTPRC*** *APOA1* *SORBS3* ***UNC13A*** ***MORC2*** ***CDK5RAP2*** *SKAP1* *TSC1* ***LDLRAD4*** ***NDUFA13*** *KIF13B* ***CLEC16A*** <u>*HDAC4*</u> *NOX4* ***MYH14*** <u>*RAPGEF1*</u> ***GATA3*** ***NEURL1*** *BVES* *DPYSL3* *PREX1* *KDM4B* ***KLK8*** *PPARG* *BCAS3* *GNA12* *DISC1* ***ATP7A*** ***PTK2*** *ADGRB1* <u>*TRAF3IP1*</u> *ITSN1* *CYFIP1* *CRMP1* ***GPM6B*** ***RNF4*** ***HNF4A*** *DNMBP* ***CDH13*** ***CTBP1*** *TRAPPC12* *INSIG1* *MYO18A* ***DYNC1H1*** ***TENM1*** ***FGF13*** *PPP1R13B*
Regulation of cellular response to growth factor stimulus	0.0102	11	277	*ADAMTS12* *NEO1* ***GATA3*** *ACVR2A* ***PRKCB*** ***LDLRAD4*** *ADAMTSL2* *CYFIP1* *EPN2* ***DSTYK*** *CITED1*
Regulation of centromere complex assembly	0.0226	2	5	***RNF4*** *TRAPPC12*
Regulation of circadian rhythm	0.0309	6	114	*NR1D2* ***HNF4A*** *THRAP3* ***MAPK10*** *PPARG* ***ZFHX3***
Regulation of cytokine biosynthetic process	0.0395	6	124	***MAP2K3*** ***PTPRC*** ***GATA3*** *INPP5D* *SIGIRR* *APP*
Regulation of cytoskeleton organization	0.0168	16	563	*APOA1* *SORBS3* *TSC1* *PRKN* *NOX4* *PREX1* ***CDK5RAP2*** *BCAS3* <u>*TRAF3IP1*</u> ***GPM6B*** ***RNF4*** ***DYNC1H1*** *CYFIP1* ***TENM1*** ***FGF13*** ***PTK2***
Regulation of developmental process	0.0047	53	2763	*APP* ***BRINP1*** ***GATA3*** ***ZFHX3*** *TNFRSF21* *NTRK1* *PPARG* ***TBX5*** ***CDK6*** *APOA1* *ACVR2A* ***UNC13A*** *ADAMTS7* *ADAMTS12* *ADGRB1* *KIF13B* <u>*HDAC4*</u> ***MLH1*** ***PTPRC*** ***HNF4A*** ***MYH14*** <u>*RAPGEF1*</u> ***NEURL1*** *BVES* *DPYSL3* *PREX1* *CITED1* ***KLK8*** ***CDK5RAP2*** *GNA12* *DISC1* ***TSHR*** *TSC1* ***PRKCB*** *INPP5D*

				PTK2 *NR1D2 PRKN RXRA INSIG1 ZNF536* <u>*TRAF3IP1*</u> *ITSN1 CYFIP1 CRMP1* ***GPM6B*** *EPN2 DNMBP FOXA2* ***WWC2 LDLRAD4 FGF13*** *RNF207*
Regulation of DNA demethylation	0.0354	2	7	*OTUD4* ***GATA3***
Regulation of embryonic development	0.0494	6	135	*APOA1* ***HNF4A GATA3*** <u>*TRAF3IP1*</u> *FOXA2 RNF207*
Regulation of Fc-gamma receptor signaling pathway involved in phagocytosis	0.0411	2	8	***PTPRC*** <u>*RAPGEF1*</u>
Regulation of filopodium assembly	0.0284	4	45	***NEURL1*** *DPYSL3 BCAS3* ***TENM1***
Regulation of gene expression	0.0430	75	4923	***RNF4*** *KAT6A* ***NLRC4 MAPK10 SUDS3*** *FOXA2 CITED1 PPARG* ***ZFHX3*** *ZNF646 PRDM10 NR1D2* ***RUVBL1*** *ETV4 SUPT3H ZNF536* <u>*ZNF407*</u> *CYFIP1* ***MAP2K3*** *THRAP3* <u>*HDAC4*</u> ***TBX5*** *RCOR1* ***HNF4A CDK6 GATA3*** *FRK IL18R1 ACVR2A NSRP1* ***MORC2 CDK5RAP2*** *SKAP1 BCAS3 SUSD4* ***WWC2*** *TSC1* ***ARNT2*** *PRKN* ***NDUFA13*** *RXRA INSIG1 NTRK1 MBTD1 NEO1 LMCD1* ***MLH1 PTPRC NEURL1*** *ZNF684 SORBS3* ***PHF20L1*** *TRIM29 APP* <u>*ADCY8*</u> *TDRD9* ***CTBP1 ATP7A DRGX PRKCB BRF1*** *SND1 MYOM1 AXIN1* ***CDH13*** *RPTOR RNF207* ***SERPINB9*** *SIGIRR* ***TENM1 MDFI*** *TNPO1* ***NUP188*** *HTATSF1* ***EIF4H***
Regulation of interleukin-5 secretion	0.0411	2	8	*TNFRSF21* ***GATA3***
Regulation of intracellular signal transduction	0.0284	38	2027	***MAP2K3 RRAGB*** *MST1R TSC1 NTRK1 APOA1* ***CDH13*** *RPTOR* ***ITPR1 WWC2*** *PRKN LMCD1* ***PTPRC*** *NOX4* <u>*RAPGEF1*</u> *DNMBP* ***MAPK10*** *IL18R1 SORBS3 PREX1 GNA12* ***PRKCB*** *ITSN1 APP* ***RANBP9 CLEC16A GATA3 CGNL1 DSTYK*** <u>*GAREM1*</u> ***PTK2 NPLOC4 NDUFA13 TENM1*** *AXIN1* ***MYO9A*** *KAT6A PPP1R13B*
Regulation of kinase activity	0.0178	22	916	***MAP2K3*** *RPTOR* ***LAT PTPRC*** *AXIN1 MST1R PRKN NOX4* ***NEURL1 FAM20A MAPK10 FGF13*** *APP TSC1 ITSN1* ***DSTYK PTK2 TENM1*** *FOXA2* <u>*ADCY8*</u> <u>*RAPGEF1*</u> *NTRK1*
Regulation of kinetochore assembly	0.0104	2	3	***RNF4*** *TRAPPC12*
Regulation of localization	0.0147	52	2905	***RUVBL1 GPM6B CACNA1I*** *APOA1 PPARG TNFRSF21* ***ITPR1*** *KCNE5* ***DHRS7C*** *PRKN EIPR1* ***PTPRC TBX5 GATA3*** *CRTAM* ***CDH13 STXBP4 PTK2*** *ADGRB1 INSIG1* <u>*HDAC4*</u> *NOX4* ***HNF4A*** <u>*RAPGEF1*</u> *P2RX1 BVES DPYSL3 PREX1* ***UNC13A*** *TRIM29 BCAS3 GNA12* <u>*ADCY8*</u> ***ATP7A PRKCB*** *CATSPER4*

				*ITSN1 CYFIP1 MYOM1 RNF207 **MAP2K3** APP **MCU LDLRAD4** TRAPPC12 MYO18A **DYNC1H1 TENM1 CDK6** FOXA2 **FGF13** PPP1R13B*
Regulation of modification of postsynaptic actin cytoskeleton	0.0300	2	6	*ITSN1 CYFIP1*
Regulation of modification of postsynaptic structure	0.0482	2	9	*ITSN1 CYFIP1*
Regulation of multicellular organismal development	0.0019	46	2138	*APP **BRINP1 GATA3 ZFHX3** TNFRSF21 NTRK1 **TBX5 CDK6** APOA1 ACVR2A **UNC13A** PPARG ADAMTS7 ADAMTS12 ADGRB1 KIF13B HDAC4 **MLH1 PTPRC HNF4A** RAPGEF1 **NEURL1** DPYSL3 PREX1 CITED1 **KLK8 CDK5RAP2** DISC1 TSC1 **PRKCB** INPP5D **PTK2** NR1D2 PRKN RXRA ZNF536 TRAF3IP1 ITSN1 CYFIP1 CRMP1 **GPM6B** EPN2 FOXA2 **LDLRAD4 FGF13** RNF207*
Regulation of multicellular organismal process	0.0062	61	3382	*APP **BRINP1 GATA3 ZFHX3** TNFRSF21 KCNE5 NTRK1 **PTPRC TBX5 CDK6** CRTAM IL18R1 APOA1 ACVR2A FOXA2 **UNC13A** PPARG ADAMTS7 ADAMTS12 ADGRB1 KIF13B **MAP2K3** HDAC4 LMCD1 **MLH1 HNF4A** RAPGEF1 **NEURL1** P2RX1 BVES DPYSL3 PREX1 CITED1 **KLK8 PEMT CDK5RAP2** BCAS3 DISC1 **TSHR** TSC1 **PRKCB** INPP5D **PTK2** NR1D2 SIGIRR PRKN RXRA **DYNC1H1** ZNF536 TRAF3IP1 ITSN1 CYFIP1 CRMP1 **GPM6B** EPN2 **WWC2** RNF207 **LDLRAD4 NPLOC4 FGF13 ITPR1***
Regulation of nervous system development	0.0031	26	953	*APP **BRINP1 ZFHX3** TNFRSF21 NTRK1 **UNC13A** KIF13B RAPGEF1 **GATA3 NEURL1** DPYSL3 PREX1 **KLK8** PPARG **CDK5RAP2** DISC1 TSC1 **PTK2** ADGRB1 PRKN ZNF536 TRAF3IP1 ITSN1 CYFIP1 CRMP1 **FGF13***
Regulation of neurogenesis	0.0031	24	844	***BRINP1 ZFHX3** NTRK1 **UNC13A** KIF13B RAPGEF1 **GATA3 NEURL1** DPYSL3 PREX1 **KLK8** PPARG **CDK5RAP2** APP TNFRSF21 DISC1 TSC1 **PTK2** PRKN ZNF536 ITSN1 CYFIP1 CRMP1 **FGF13***
Regulation of neuron differentiation	0.0013	22	665	***BRINP1 ZFHX3** NTRK1 **UNC13A** KIF13B RAPGEF1 **GATA3 NEURL1** DPYSL3 PREX1 **KLK8 CDK5RAP2** APP DISC1 TSC1 **PTK2** PRKN ZNF536 ITSN1 CYFIP1 CRMP1 **FGF13***
Regulation of neuron projection development	0.0043	17	506	*NTRK1 **UNC13A** KIF13B RAPGEF1 **GATA3 NEURL1** DPYSL3 PREX1 **KLK8** DISC1 TSC1 **PTK2** PRKN ITSN1 CYFIP1 CRMP1 **FGF13***

Regulation of nucleobase-containing compound metabolic process	0.0482	69	4482	***RNF4*** *KAT6A* ***NLRC4*** ***SUDS3*** *FOXA2* *CITED1* *PPARG* ***ZFHX3*** *ZNF646* *NR1D2* ***RUVBL1*** *ETV4* *SUPT3H* *ZNF536* ***MAP2K3*** *THRAP3* <u>*HDAC4*</u> ***TBX5*** *RCOR1* ***HNF4A*** ***GATA3*** *FRK* *IL18R1* *ACVR2A* *NSRP1* ***MORC2*** ***CDK5RAP2*** *SKAP1* *BCAS3* ***WWC2*** *OTUD4* ***ARNT2*** *PRKN* ***NDUFA13*** *RXRA* *INSIG1* *NTRK1* *MBTD1* *NEO1* *LMCD1* ***MLH1*** ***PTPRC*** *NOX4* ***CDK6*** ***MAPK10*** *ZNF684* *SORBS3* ***PHF20L1*** *TRIM29* *APP* <u>*ADCY8*</u> ***CTBP1*** ***DRGX*** ***PRKCB*** *PRDM10* ***BRF1*** *SND1* <u>*ZNF407*</u> *AXIN1* ***CDH13*** *RPTOR* *SIGIRR* ***TENM1*** ***MDFI*** ***ATP7A*** *TNPO1* ***NUP188*** *HTATSF1* *POLH*
Regulation of peptidyl-threonine phosphorylation	0.0284	4	45	*PPP2R5D* *RPTOR* *APP* *AXIN1*
Regulation of phosphorylation	0.0226	33	1653	***MAP2K3*** *PPP2R5D* *RPTOR* *PRKN* *NTRK1* ***LAT*** ***PTPRC*** *AXIN1* ***FAM20A*** *APP* *MST1R* <u>*HDAC4*</u> *NOX4* <u>*RAPGEF1*</u> ***NEURL1*** ***MAPK10*** *APOA1* *SORBS3* ***FGF13*** *TSC1* *ITSN1* ***PTK2*** ***RANBP9*** *ACVR2A* ***DSTYK*** <u>*GAREM1*</u> ***LDLRAD4*** <u>*TRAF3IP1*</u> ***TENM1*** *FOXA2* <u>*ADCY8*</u> ***ATP7A*** ***NUP188***
Regulation of plasma membrane bounded cell projection organization	0.0031	21	682	*NTRK1* ***UNC13A*** *KIF13B* <u>*HDAC4*</u> <u>*RAPGEF1*</u> ***GATA3*** ***NEURL1*** *DPYSL3* *PREX1* ***KLK8*** *BCAS3* *DISC1* ***ATP7A*** *TSC1* ***PTK2*** *PRKN* *ITSN1* *CYFIP1* *CRMP1* ***TENM1*** ***FGF13***
Regulation of protein metabolic process	0.0340	51	3006	***MAP2K3*** ***NLRC4*** *PPP2R5D* *RPTOR* *OTUD4* ***SERPINB9*** *PRKN* *NTRK1* ***LAT*** *CYFIP1* *AXIN1* ***NDUFA13*** <u>*HDAC4*</u> ***PTPRC*** ***FAM20A*** *PPARG* *APP* *SUSD4* *MST1R* *TSC1* ***CLEC16A*** *NOX4* <u>*RAPGEF1*</u> ***GATA3*** ***NEURL1*** *P2RX1* ***MAPK10*** *APOA1* *SORBS3* *KDM4B* ***FGF13*** ***UNC13A*** ***PEMT*** *GNA12* *DISC1* <u>*COL6A3*</u> *INPP5D* ***PTK2*** *ADGRB1* *SIGIRR* *RXRA* ***RANBP9*** *ACVR2A* ***DSTYK*** <u>*GAREM1*</u> ***CTBP1*** ***LDLRAD4*** <u>*TRAF3IP1*</u> ***TENM1*** <u>*ADCY8*</u> ***EIF4H***
Regulation of response to stimulus	0.0018	84	4820	***MAP2K3*** ***RRAGB*** ***NLRC4*** ***NEURL1*** *MST1R* *OTUD4* *TSC1* ***CSNK1G1*** *PRKN* *PEAR1* *NTRK1* *DENND1B* *AXIN1* *APP* ***PTPRC*** ***CDK6*** *CRTAM* *IL18R1* *APOA1* *ACVR2A* *FOXA2* ***CDH13*** *SKAP1* *RPTOR* *SUSD4* ***ITPR1*** *ADAMTS12* ***WWC2*** ***STXBP4*** ***LDLRAD4*** ***RUVBL1*** ***IFNLR1*** *INSIG1* *NEO1* <u>*HDAC4*</u> *LMCD1* ***MLH1*** *NOX4* <u>*RAPGEF1*</u> ***GATA3*** *DNMBP* ***MAPK10*** *SORBS3* *PREX1* ***KLK8*** ***FGF13*** ***UNC13A*** *PPARG* *TNFRSF21* *GNA12* <u>*ADCY8*</u> *DISC1* ***ATP7A*** ***PRKCB*** *INPP5D* ***PTK2*** *NR1D2* *SIGIRR* *ADAMTSL2* *ZNF536* <u>*TRAF3IP1*</u> *ITSN1* ***LAT*** *CYFIP1* ***RANBP9*** ***CLEC16A*** *EPN2* ***CGNL1*** ***DSTYK*** <u>*GAREM1*</u> ***SERPINB9*** ***NPLOC4***

				NDUFA13 TENM1 MDFI *CD99* ***MYO9A*** *KAT6A PPP1R13B* ***NUP188*** *CITED1 CTSB* ***POLH GRK5***
Regulation of retrograde transport. endosome to Golgi	0.0162	2	4	*EIPR1 PRKN*
Regulation of signal transduction	0.0043	64	3529	***MAP2K3 RRAGB NEURL1*** *MST1R OTUD4 TSC1* ***CSNK1G1*** *PEAR1 NTRK1 AXIN1 APP APOA1 ACVR2A* ***CDH13*** *RPTOR* ***ITPR1*** *ADAMTS12* ***WWC2 LDLRAD4 RUVBL1*** *PRKN INSIG1 NEO1 LMCD1* ***PTPRC*** *NOX4 RAPGEF1* ***GATA3*** *DNMBP* ***MAPK10*** *IL18R1 SORBS3 PREX1* ***FGF13 UNC13A*** *SKAP1 GNA12 DISC1* ***PRKCB*** *INPP5D* ***PTK2*** *SIGIRR ADAMTSL2 ZNF536 TRAF3IP1 ITSN1* ***LAT*** *CYFIP1* ***RANBP9 CLEC16A*** *EPN2* ***CGNL1*** *PPARG* ***DSTYK*** *GAREM1* ***NPLOC4 NDUFA13 TENM1 MDFI MYO9A*** *KAT6A PPP1R13B CITED1* ***GRK5***
Regulation of signaling	0.0007	75	3952	***MAP2K3 RRAGB NEURL1 GRIK3*** *MST1R OTUD4 TSC1* ***CSNK1G1*** *GRID1 PEAR1 NTRK1 AXIN1 APP APOA1 ACVR2A* ***CDH13*** *RPTOR* ***ITPR1*** *ADAMTS12* ***WWC2 STXBP4 LDLRAD4 RUVBL1*** *PRKN INSIG1 NEO1 LMCD1* ***PTPRC*** *NOX4* ***HNF4A*** *RAPGEF1* ***GATA3*** *DNMBP P2RX1* ***MAPK10*** *BVES IL18R1 SORBS3 PREX1* ***FGF13 UNC13A*** *SKAP1 GNA12 ADCY8 DISC1* ***PRKCB*** *INPP5D* ***PTK2*** *ADGRB1 SIGIRR ADAMTSL2 ZNF536 TRAF3IP1 ITSN1* ***LAT*** *CYFIP1* ***RANBP9 CLEC16A*** *EPN2* ***CGNL1*** *PPARG* ***DSTYK*** *GAREM1* ***MCU NPLOC4 NDUFA13 TENM1 MDFI*** *FOXA2 RNF207* ***MYO9A*** *KAT6A PPP1R13B CITED1* ***GRK5***
Regulation of supramolecular fiber organization	0.0437	11	372	*APOA1 SORBS3 TSC1 PRKN NOX4 PREX1* ***CDK5RAP2*** *APP CYFIP1* ***TENM1 FGF13***
Regulation of synapse organization	0.0292	9	239	*APP* ***NEURL1 KLK8*** *DISC1* ***PTK2*** *ADGRB1 NTRK1 ITSN1 CYFIP1*
Regulation of synapse structure or activity	0.0339	9	248	*APP* ***NEURL1 KLK8*** *DISC1* ***PTK2*** *ADGRB1 NTRK1 ITSN1 CYFIP1*
Regulation of transcription by RNA polymerase III	0.0331	3	23	***BRF1*** *RPTOR* ***TENM1***
Regulation of transferase activity	0.0275	23	1021	***MAP2K3*** *RPTOR* ***LAT PTPRC*** *AXIN1 MST1R PRKN NOX4* ***NEURL1 FAM20A MAPK10 FGF13*** *PPARG APP TSC1 ITSN1* ***DSTYK PTK2 TENM1*** *FOXA2 ADCY8 RAPGEF1 NTRK1*

Regulation of transport	0.0336	37	1991	**GPM6B** **CACNA1I** APOA1 TNFRSF21 KCNE5 **DHRS7C** PPARG EIPR1 **PTPRC** **GATA3** CRTAM **STXBP4** PRKN INSIG1 **HNF4A** RAPGEF1 P2RX1 BVES **UNC13A** BCAS3 ADCY8 **ATP7A** **PRKCB** CATSPER4 ITSN1 CYFIP1 MYOM1 RNF207 **CDH13** **MCU** MYO18A **DYNC1H1** **TENM1** FOXA2 PPP1R13B APP **ITPR1**
Regulation of trans-synaptic signaling	0.0392	13	468	**GRIK3** GRID1 **NEURL1** P2RX1 **UNC13A** ADCY8 DISC1 **PRKCB** ADGRB1 PRKN NTRK1 CYFIP1 APP
Response to acid chemical	0.0393	11	360	**BRINP1** **RRAGB** PPARG RPTOR **NDUFA13** **NEURL1** **MAPK10** **PEMT** **ATP7A** NTRK1 RXRA
Response to axon injury	0.0284	5	75	DPYSL3 MTR APOA1 **KLK8** NTRK1
Response to endogenous stimulus	0.0014	40	1704	**RRAGB** ACVR2A PPARG RPTOR **TSHR** TSC1 NR1D2 NTRK1 RAPGEF1 CITED1 **DSTYK** ADAMTS7 **ZFHX3** **CDH13** GAREM1 ADAMTS12 **PTK2** NEO1 HDAC4 NOX4 **HNF4A** **GATA3** **NEURL1** APP ADCY8 **ATP7A** **STXBP4** **LDLRAD4** **ARNT2** RXRA ADAMTSL2 CYFIP1 CTSB SKAP1 **PRKCB** **RNF4** AXIN1 ATP6V0D2 PRKN CATSPER4
Response to estrogen	0.0082	6	77	CITED1 BCAS3 APOA1 PPARG **GATA3** **SERPINB9**
Response to external stimulus	0.0049	50	2561	**RRAGB** **EFNB1** **NLRC4** BCAS3 RPTOR CYFIP1 APOA1 FOXA2 **CDH13** SUSD4 ADAMTS12 MST1R **SERPINB9** **IFNLR1** NEO1 HDAC4 CRMP1 **MLH1** **PTPRC** **ATG16L1** **GATA3** P2RX1 **FAM20A** PREX1 CITED1 LOXL1 **KLK8** PPARG **PEMT** APP **ITGA9** **DPYSL4** **NFASC** **DRGX** TSC1 NR1D2 NAALADL2 DEFB123 ADGRB1 PRKN NTRK1 TRAF3IP1 **CDK6** **CLEC16A** **NPLOC4** **LYST** **RANBP9** **ATP7A** **PTK2** SIGIRR
Response to growth factor	0.0028	22	723	ACVR2A NTRK1 RAPGEF1 CITED1 **DSTYK** ADAMTS7 **ZFHX3** GAREM1 ADAMTS12 **PTK2** NEO1 NOX4 **GATA3** APP **ATP7A** **PRKCB** **LDLRAD4** ADAMTSL2 CYFIP1 **MAP2K3** EPN2 SKAP1
Response to manganese ion	0.0284	3	21	APP **ATP7A** PRKN
Response to organic substance	0.0034	65	3547	**BRINP1** **RRAGB** **MAPK10** ACVR2A PPARG RPTOR OTUD4 **TSHR** TSC1 NR1D2 **IFNLR1** NTRK1 RAPGEF1 IL18R1 APOA1 CITED1 **DSTYK** ADAMTS7 **ZFHX3** **CDH13** GAREM1 TNFRSF21 ADAMTS12 **STXBP4** **PTK2** PRKN **NDUFA13** INSIG1 NEO1 HDAC4 **PTPRC** NOX4 **HNF4A** **GATA3** **NEURL1** P2RX1 LOXL1 **PEMT** APP ADCY8 MST1R **ATP7A** **PRKCB**

				LDLRAD4 ***ARNT2*** *SIGIRR* *RXRA* ***IL17REL*** *ADAMTSL2* *CYFIP1* *CTSB* ***MAP2K3*** *EPN2* *SKAP1* ***NPLOC4*** *DPYSL3* *FOXA2* ***RNF4*** *ACADVL* *KLHL20* *AXIN1* *PTPN5* *ATP6V0D2* *INPP5D* *CATSPER4*
Response to organonitrogen compound	0.0411	22	1019	***RRAGB*** *PPARG* *RPTOR* *TSC1* <u>*RAPGEF1*</u> ***CDH13*** ***PTK2*** *NOX4* ***NEURL1*** *P2RX1* *CITED1* ***PEMT*** *APP* <u>*ADCY8*</u> ***ATP7A*** ***STXBP4*** *CYFIP1* ***TSHR*** ***NPLOC4*** ***PRKCB*** *PRKN* *ATP6V0D2*
Response to oxygen-containing compound	0.0233	34	1725	***BRINP1*** ***RRAGB*** *PPARG* *RPTOR* *TSC1* <u>*RAPGEF1*</u> ***STXBP4*** ***PTK2*** ***NDUFA13*** *INSIG1* *NOX4* ***HNF4A*** ***GATA3*** ***NEURL1*** *P2RX1* ***MAPK10*** *MTR* *CITED1* *LOXL1* ***PEMT*** *APP* <u>*ADCY8*</u> ***ATP7A*** ***PRKCB*** ***ARNT2*** *NTRK1* *CYFIP1* ***TSHR*** *RXRA* *FOXA2* *ATP6V0D2* *SIGIRR* *PRKN* *CATSPER4*
Skeletal muscle organ development	0.0499	7	180	*BVES* *MYOM2* <u>*HDAC4*</u> *MYOM1* ***NEURL1*** *NR1D2* ***MYH14***
Skeletal muscle tissue development	0.0420	7	171	*BVES* *MYOM2* <u>*HDAC4*</u> *MYOM1* ***NEURL1*** *NR1D2* ***MYH14***
Supramolecular fiber organization	0.0138	19	712	*MYOM2* *MYOM1* *PLOD2* ***DYNC1H1*** *APOA1* *SORBS3* *TSC1* *PRKN* *NOX4* *DPYSL3* *PREX1* ***CGNL1*** ***CDK5RAP2*** *APP* *CYFIP1* ***TENM1*** ***FGF13*** ***ATP7A*** ***RANBP9***
Synapse organization	0.0482	12	434	*APP* ***UNC13A*** *CYFIP1* ***NEURL1*** ***KLK8*** *DISC1* ***NFASC*** *TSC1* ***PTK2*** *ADGRB1* *NTRK1* *ITSN1*
Synaptic transmission. glutamatergic	0.0068	7	103	***UNC13A*** ***GRIK3*** *GRID1* *P2RX1* *DISC1* *PRKN* *NTRK1*
Taxis	0.0340	17	683	***EFNB1*** *CYFIP1* *APOA1* ***CDH13*** *NEO1* *CRMP1* ***GATA3*** *PREX1* *APP* ***ITGA9*** ***DPYSL4*** ***NFASC*** ***DRGX*** *NTRK1* ***LYST*** ***RANBP9*** ***PTK2***
TOR signaling	0.0125	7	121	***RRAGB*** *RPTOR* *TSC1* ***GATA3*** *GNA12* *DISC1* ***CLEC16A***
Transmembrane receptor protein tyrosine kinase signaling pathway	0.0087	20	730	***EFNB1*** *FRK* *MST1R* *TSC1* ***PTK2*** *NTRK1* <u>*GAREM1*</u> *ADAMTS12* <u>*RAPGEF1*</u> ***GATA3*** ***NEURL1*** *APP* ***STXBP4*** ***PRKCB*** *CYFIP1* *EPN2* ***DSTYK*** ***CDH13*** *ATP6V0D2* *ITSN1*
Vesicle-mediated transport	0.0354	40	2220	***CLEC16A*** ***SEC31B*** *KLHL20* ***CACNA1I*** ***LMAN2L*** ***UNC13A*** ***CSNK1G1*** *ADGRB1* *DENND1B* *EIPR1* ***PTPRC*** *APOA1* *PRKN* *EPN2* *SCAMP1* <u>*RAPGEF1*</u> *P2RX1* ***MAPK10*** *BVES* *PPARG* *APP* *SYN2* ***PRKCB*** *TRAPPC12* *PEAR1* *ITSN1* ***LAT*** ***CDH13*** ***LYST*** *INSIG1* *MYO18A* *ACAA1* ***KIFAP3*** *FRK* ***C1orf35*** ***NFASC*** *CTSB* ***PTK2*** ***DYNC1H1*** *CYFIP1*

[1]Number of genes differentially methylated in the functional category

[2]Hypermethylated genes in the F1-RES group are marked in bold.

[3]Genes showing both, hyper- and hypo-methylated sites are underlined

Supplementary Table 4. Enriched GO terms (TSS) from the list of differentially methylated genes in the of early feed restricted ewes (F0-RES).

Functional category	Enrichment FDR	Genes in list[1]	Total genes	Genes symbol[2]
Regulation of collagen metabolic process	0.0243	2	41	***F2 SCX***
Positive regulation of collagen metabolic process	0.0243	2	27	***F2 SCX***
Collagen biosynthetic process	0.0243	2	45	***F2 SCX***
Regulation of collagen biosynthetic process	0.0243	2	36	***F2 SCX***
Positive regulation of collagen biosynthetic process	0.0243	2	26	***F2 SCX***
Immune effector process	0.0243	6	1392	***PMAIP1 SUSD4 F2*** *FUT7 LAMP1* ***DOCK1***
Positive regulation of immune response	0.0301	5	991	*BCAR1* ***SUSD4*** *LAMP1* ***DOCK1 F2***
Regulation of protein activation cascade	0.0402	2	71	***SUSD4 F2***
Regulation of complement activation	0.0402	2	70	***SUSD4 F2***
Complement activation	0.0483	2	93	***SUSD4 F2***
Activation of immune response	0.0483	4	763	*BCAR1* ***SUSD4 DOCK1 F2***
Positive regulation of immune system process	0.0483	5	1301	*BCAR1* ***SUSD4*** *LAMP1* ***DOCK1 F2***
Vascular endothelial growth factor receptor signaling pathway	0.0483	2	92	*BCAR1* ***DOCK1***
Regulation of immune response	0.0483	5	1325	*BCAR1* ***SUSD4*** *LAMP1* ***DOCK1 F2***
Protein maturation	0.0483	3	376	***GLRX3 SUSD4 F2***
Regulation of humoral immune response	0.0483	2	89	***SUSD4 F2***

[1]Number of genes differentially methylated in the functional category
[2]Hypermethylated genes in the F0-RES group are marked in bold.

Supplementary Table 5. Chromatographic and mass spectrum data for the selected metabolites in each method. $(PA)_S/\Sigma(PA)_S$, where PA is the chromatographic peak area for a given m/z in each sample, is given as mean ± standard deviation.

**Metabolite**	**Method**	**Rt (min)**	**m/z**	**Ion**	**$(PA)_S/\Sigma(PA)_S$ ($\times10^{-2}$)**	
					**ADL**	**RES**
(Homo)2-citrate	1	0.68	203.0547	$[M-H_2O+H]^+$	3.81 ± 0.23	4.46 ± 0.65
Glycodeoxycolate	1	2.99	432.3106	$[M-H_2O+H]^+$	3.49 ± 2.80	4.73 ± 5.51
Ketodeoxycholic acid	1	2.97	373.2743	$[M-H_2O+H]^+$	4.47 ± 3.06	3.91 ± 4.14
	2	3.28	373.2743	$[M-H_2O+H]^+$	4.46 ± 3.73	3.92 ± 4.11
Cholic acid	1	2.98	426.3217	$[M+NH_4]^+$	4.44 ± 2.04	3.94 ± 3.25
	2	3.28			5.15 ± 4.65	3.33 ± 2.97
Hydroxy-cholen-24-oic acid	1	3.43	357.2791<br>375.2912	$[M-H_2O+H]^+$<br>$[M+H]^+$	8.92 ± 7.26	0.14 ± 0.38
Dihydroxy-cholen-24-oic acid	1	2.98	391.2843	$[M+H]^+$	4.61 ± 2.69	3.79 ± 3.40
Glycocholic acid	1	2.69	430.2958	$[M-2H_2O+H]^+$	3.69 ± 2.66	4.54 ± 5.43
	2	2.95	466.3164	$[M+H]^+$	3.35 ± 2.26	4.86 ± 5.70
Triethanolamine (Trolamine)	1	1.14	132.1027	$[M-H_2O+H]^+$	3.90 ± 1.04	4.39 ± 1.23
Indolelactic acid	1	2.09	188.0721	$[M-H_2O+H]^+$	4.02 ± 0.78	4.29 ± 0.73
Tryptophan	1	2.09	205.0979	$[M+H]^+$	4.17 ± 0.50	4.16 ± 0.81
C-10 Isoprenoid	2	2.49	183.1023	$[M+H]^+$	5.11 ± 3.79	3.36 ± 3.43
Taurocholic acid 3-sulfate	2	5.83	289.6494	$[M-H_2O+2H]^{+2}$	5.18 ± 3.30	3.30 ± 1.28
Taurodeoxycholic acid	2	3.63	500.3022	$[M+H]^+$	2.79 ± 1.98	5.36 ± 3.37
Taurocholic acid	2	3.10	516.2988	$[M+H]^+$	2.95 ± 2.01	5.18 ± 3.26
LPC(17:1)	2	4.19	508.3379	$[M+H]^+$	4.07 ± 1.60	4.25 ± 1.66
LPC(18:3)	2	3.57	518.3242	$[M+H]^+$	3.99 ± 1.65	4.32 ± 1.27
LPC(18:3)	2	4.23	518.3242	$[M+H]^+$	4.24 ± 1.27	4.11 ± 1.01
LPC(18:1)	2	4.44	522.3545	$[M+H]^+$	3.30 ± 1.90	4.89 ± 2.21
LPC(22:5)	2	4.07	570.3551	$[M+H]^+$	3.99 ± 0.77	4.31 ± 0.69
LPC(22:4)	2	4.68	572.3701	$[M+H]^+$	4.01 ± 0.67	4.30 ± 1.34
LPC(18:0)	2	5.79	524.3709	$[M+H]^+$	3.82 ± 0.89	4.46 ± 0.85
1-hexadecanoyl-2-succinyl-sn-glycero-3-phosphate (PS-PA)	2	4.00	528.2896	$[M+H]^+$	5.11 ± 3.30	3.33 ± 0.89

Supplementary Figure 1. Correlation plots of DNA methylation patterns for F0 and F1 samples, respectively.

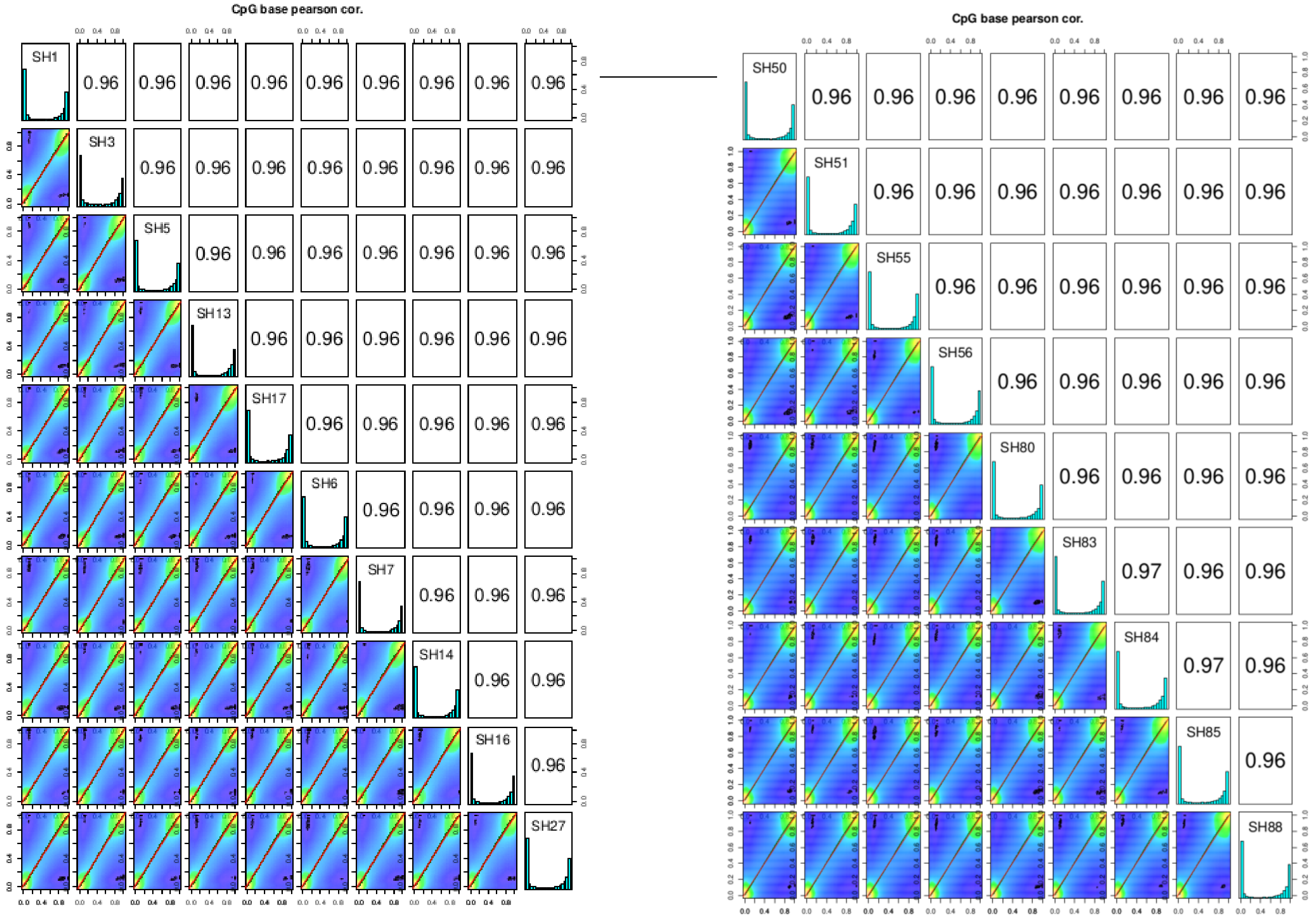

Supplementary Figure 2. Typical chromatograms and averaged mass spectra obtained in the UPLC-MS analysis. (A) and (B) method 1 of positive ionization mode; (C) and (D) negative ionization mode; and (E) and (F) method 2 of positive ionization mode.

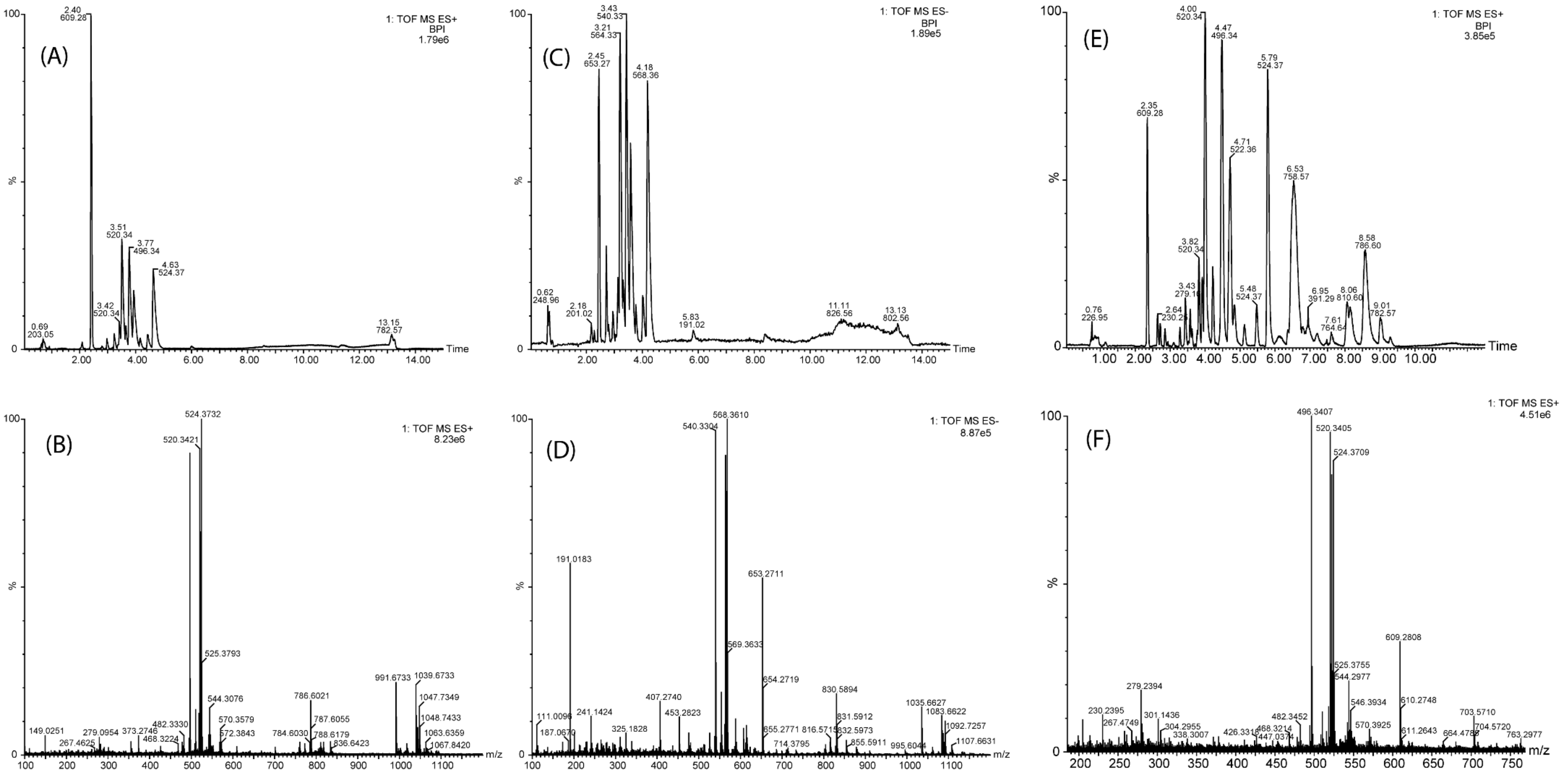

Supplement: Supplementary file 1 [file Presentation_1.pdf]
